# WRKY Transcription Factors: Integral Regulators of Defence Responses to Biotic Stress in Crops

**DOI:** 10.1111/pbi.70542

**Published:** 2026-01-12

**Authors:** Dongjiao Wang, Ruize Zhang, Wenhui Zou, Yuanyuan Zhang, Wanying Zhao, Tingting Sun, Qibin Wu, Zheng Qing Fu, Youxiong Que

**Affiliations:** ^1^ State Key Laboratory for Tropical Crop Breeding, Institute of Tropical Bioscience and Biotechnology, Sanya Research Institute Chinese Academy of Tropical Agricultural Sciences Sanya Hainan China; ^2^ Department of Biological Sciences University of South Carolina Columbia South Carolina USA; ^3^ Key Laboratory of Sugarcane Biology and Genetic Breeding, Ministry of Agriculture and Rural Affairs, National Engineering Research Center for Sugarcane, College of Agriculture Fujian Agriculture and Forestry University Fuzhou China

**Keywords:** biotic stresses, crop immunity, defence‐growth equilibrium, transcriptional regulation, WRKY transcription factors

## Abstract

Crops are continually challenged by biotic stresses, including fungal, bacterial and viral pathogens and insect pests, which cause substantial yield and quality losses worldwide. WRKY transcription factors constitute a plant‐specific and functionally diverse family that is central to immune regulation. Recent advances in genomic resources and multi‐omics approaches have accelerated the identification and functional characterisation of WRKYs in crops. This review summarises the structural features and classification of *WRKY* genes and their genome‐wide distribution across crop species. It also synthesises WRKY‐centred regulatory modules that mediate resistance to major classes of biotic stress. In antifungal defence, WRKYs reinforce pattern‐ and effector‐triggered immunity, modulate protein stability and reprogramme secondary metabolism. In antibacterial immunity, they link bacterial perception to cell wall remodelling and hormone and redox signalling. WRKYs also activate *PR* gene expression, cell wall fortification, RNA interference and programmed cell death to combat oomycete and viral pathogens and insect pests. Overall, WRKYs function as context‐dependent transcriptional hubs. They integrate immune signalling with hormonal crosstalk, remodel defence gene networks, and redirect secondary metabolism, thereby shaping resistance outcomes under biotic stress. The review examines WRKY‐mediated defence–growth trade‐offs and explores opportunities to harness WRKY‐centred networks for breeding and engineering broad‐spectrum, durable disease and pest resistance. It also highlights how integrating multi‐omics with precision genome editing, synthetic biology, gene‐drive technologies and artificial intelligence could establish WRKYs as central molecular targets for improving crop resilience and performance.

## 1 Introduction

Crops are increasingly challenged by complex and severe biotic stresses that threaten both yield and quality, especially in the context of accelerating climate change and intensifying agricultural practices (Jahan et al. 2025; Rivero et al. 2022; Zandalinas et al. 2021). Pathogenic organisms such as fungi, bacteria and viruses, and pests damage plant tissues, disrupt physiological metabolism and trigger defence responses that consume a lot of energy (He et al. 2022; Lai and Wang 2025; Wu et al. 2024; Xie et al. 2025). They result in a significant reduction in the production of vital food crops like rice ( *Oryza sativa* ), wheat ( *Triticum aestivum* ) and maize ( *Zea mays* ), with average losses calculated to be between 21.5% and 30% (Hu et al. 2024; Rezaei et al. 2023; Savary et al. 2019). They also affect economic crops such as sugarcane (*Saccharum* spp.), cotton ( *Gossypium hirsutum* ) and rapeseed ( *Brassica napus* ), often slashing yields by 10%–70% (Mehdi et al. 2024; Zheng et al. 2020). The rise in global temperatures and altered farming practices contribute to pest surges, highlighting the urgent need for adaptive stress management strategies (Hussain et al. 2023; Ma, Wang, et al. 2025; Zandalinas and Mittler 2022). For this reason, decoding how crop immunity functions at a molecular level and developing plant varieties with long‐lasting and broad‐spectrum resistance is now a main focus in plant science and breeding.

During evolution, plants have established a two‐tiered immune system to detect and defend against pathogen infection (Jones et al. 2024; Qiao et al. 2021; Roussin‐Léveillée et al. 2024; Sharma et al. 2023). When immune responses activate, transcription factors (TFs) ensure defence‐related genes are properly expressed through binding to specific DNA regions known as TF binding sites (Reboledo et al. 2022; Meshi and Iwabuchi 1995; Strader et al. 2022; Todeschini et al. 2014). Among plant‐specific TF families, WRKY TFs are recognised as core regulators of defence responses to biotic stresses (Yang, Fang, et al. 2025). To regulate gene expression, these proteins bind to W‐box *cis*‐elements, working as activators or repressors of their target genes (Ramos et al. 2023). WRKY TFs participate in multiple levels of immune regulation, including pattern‐triggered immunity (PTI) and effector‐triggered immunity (ETI) (Ramos et al. 2023). Upon pathogen challenge, they modulate the expression of key immune components like pathogenesis‐related proteins, lignin and callose biosynthesis enzymes and genes involved in oxidative bursts and secondary metabolism (Choi et al. 2020; Meng et al. 2024; Wang, Wang, et al. 2023; Xie et al. 2021) to maintain immune homeostasis.

We present here a comprehensive overview of WRKY TFs, covering their structural features, classification and regulatory roles in biotic stress responses in major crops. The review delves into recent findings about their molecular activities and functional relationships, particularly discoveries from the past 5 years, while acknowledging significant older studies. This review also explores the potential applications of WRKYs in crop improvement, aiming to support the development of crop varieties with broad‐spectrum and durable resistance through molecular breeding.

## 2 Discovery, Structure, and Classification of WRKY TFs

The WRKY family, representing a major class of plant‐specific TFs, plays a crucial role in the transcriptional regulatory networks of plants (Jiang et al. 2017). In 1994, *Sweet Potato Factor 1* (*SPF1*) was first cloned from sweet potato ( *Ipomoea batatas* ) (Ishiguro and Nakamura 1994). The encoded protein could specifically bind to SP8 in the promoters of two sporamin genes and one β‐amylase gene in sweet potato, providing early evidence for regulatory roles of WRKY‐related TFs (Ishiguro and Nakamura 1994). Subsequently, Rushton et al. isolated three DNA‐binding proteins from parsley ( *Petroselinum crispum* ), named WRKY1, WRKY2 and WRKY3, which specifically bind to W‐box elements (TTGACC/T) in the promoter of the pathogenesis‐related gene *PR1* (Rushton et al. 1996). This binding is essential for activating *PR1* expression and triggering defence responses against pathogens. It was the first to delineate the characteristic features of the WRKY TF family, including the conserved WRKYGQK motif and a zinc finger domain, thus establishing WRKYs as a plant‐specific and immune‐responsive family of TFs. The RRS1‐R protein in *Arabidopsis thaliana* is a nucleotide‐binding, leucine rich repeat (NB‐LRR) receptor that features a WRKY DNA‐binding domain fused at its C‐terminus (Sarris et al. 2015). This WRKY domain, which plays a significant role in pathogen recognition, is a crucial component of the RRS1‐R protein structure. Interestingly, RRS1‐R can form an immune receptor complex with another NB‐LRR protein, RPS4, utilising the WRKY domain as an integrated decoy to recognise the bacterial effector protein AvrRps4 or PopP2.

This interaction, in turn, initiates plant immune responses, underscoring the critical role of the WRKY domain in pathogen detection and immune system activation. Members of the WRKY gene family are characterised by the presence of one or two highly conserved WRKY domains (Yamasaki et al. 2005). These domains, each comprising approximately 60 amino acids, typically feature a conserved WRKYGQK motif at the N‐terminus, which may undergo specific amino acid substitutions in certain plant species, leading to variants such as WRKYGEK, WRKYGKK, WSKYEQK, and WRKYSEK (Zhang and Wang 2005). In addition, point mutations have been reported in the core residues, generating alternative motifs including WRRY, WSKY, WKRY, WVKKY, WRIC, WRMC, and WIKY (Jiang et al. 2017). Interestingly, the C‐terminal region of the WRKY domain is accompanied by a characteristic zinc‐finger motif, mainly the C_2_H_2_ type (CX_4–5_CX_22–23_HXH) or the C_2_HC type (CX_7_CX_23_HXC) (Xu et al. 2019). Beyond the typical WRKY domain, these proteins frequently possess additional structures associated with TFs, including basic nuclear localization signals (NLS), oligomerization sites, and transcriptional regulatory domains (Deslandes et al. 2002). Notably, some WRKY proteins are known to contain leucine zipper motifs, which promote the pairing of proteins and subsequently regulate transcriptional activation via protein interactions (Deslandes et al. 2002). These structural features work together to establish a flexible system enabling the broad functional and regulatory roles of WRKY TFs in plants.

**FIGURE 1:**
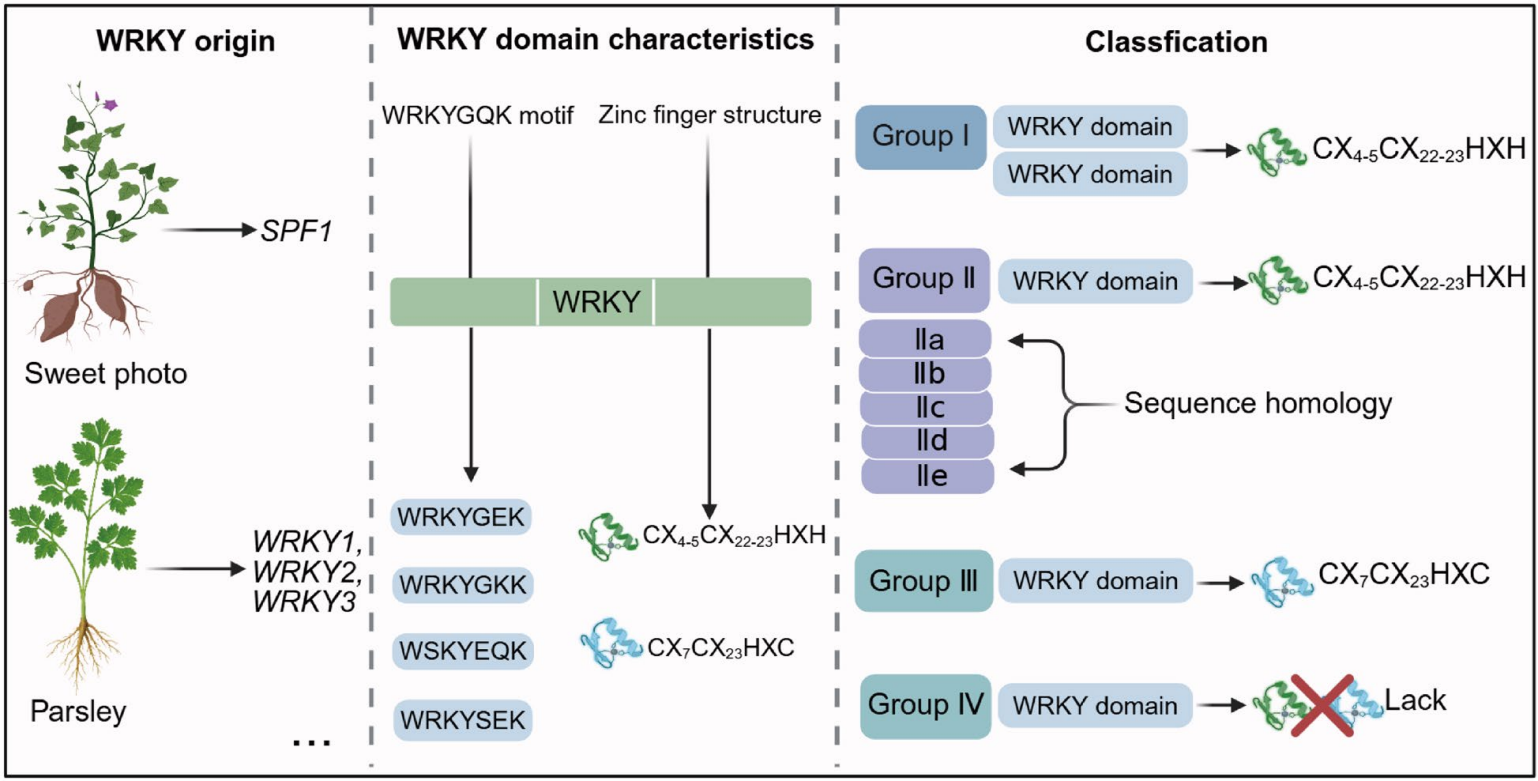
Discovery, structures and classification of WRKY transcription factors in plants. WRKY transcription factors were first identified in sweet potato (SPF1) and later characterised in parsley as WRKY1, WRKY2, and WRKY3. WRKY TFs contain a conserved WRKY domain, defined by a heptapeptide sequence (WRKYGQK) and an associated zinc finger motif. Variants in the core motif (WRKYGEK, WRKYGKK, WSKYEQK, and WRKYSEK) have also been reported. Based on the number of WRKY domains and the type of zinc finger structure, WRKYs are classified into four major groups: Group I (two WRKY domains with a CX_4‐5_CX_22–23_HXH‐type zinc finger), Group II (one WRKY domain with the same zinc finger type as Group I, further divided into subgroups IIa–IIe), Group III (one WRKY domain with a CX_7_CX_23_HXC‐type zinc finger) and Group IV (members lacking a complete zinc finger motif). This classification reflects both structural and evolutionary divergence within the WRKY family. The parsley illustration was created with the assistance of AI software (ChatGPT).

According to the number of WRKY domains, WRKY TFs are classified into three main groups. Group I features two WRKY domains, whereas Group II and III each possess a single domain (Eulgem et al. 2000). Based on sequence homology, Group II is further divided into five subgroups: IIa, IIb, IIc, IId, and IIe (Eulgem et al. 2000). Remarkably, Group I and II have a shared C_2_H_2_‐type zinc‐finger motif, defined as CX_4–5_CX_22–23_HXH, while Group III proteins are characterised by a different C_2_HC‐type motif, CX_7_CX_23_HXC (Eulgem et al. 2000). In the case of Group IV, this subset of WRKY proteins retains the WRKY heptapeptide but lacks the zinc‐finger domain (Xu et al. 2016). The structural features and classification framework of WRKY TFs are summarised in Figure 1.

## 3 WRKY Gene Family in Major Crops

**FIGURE 2:**
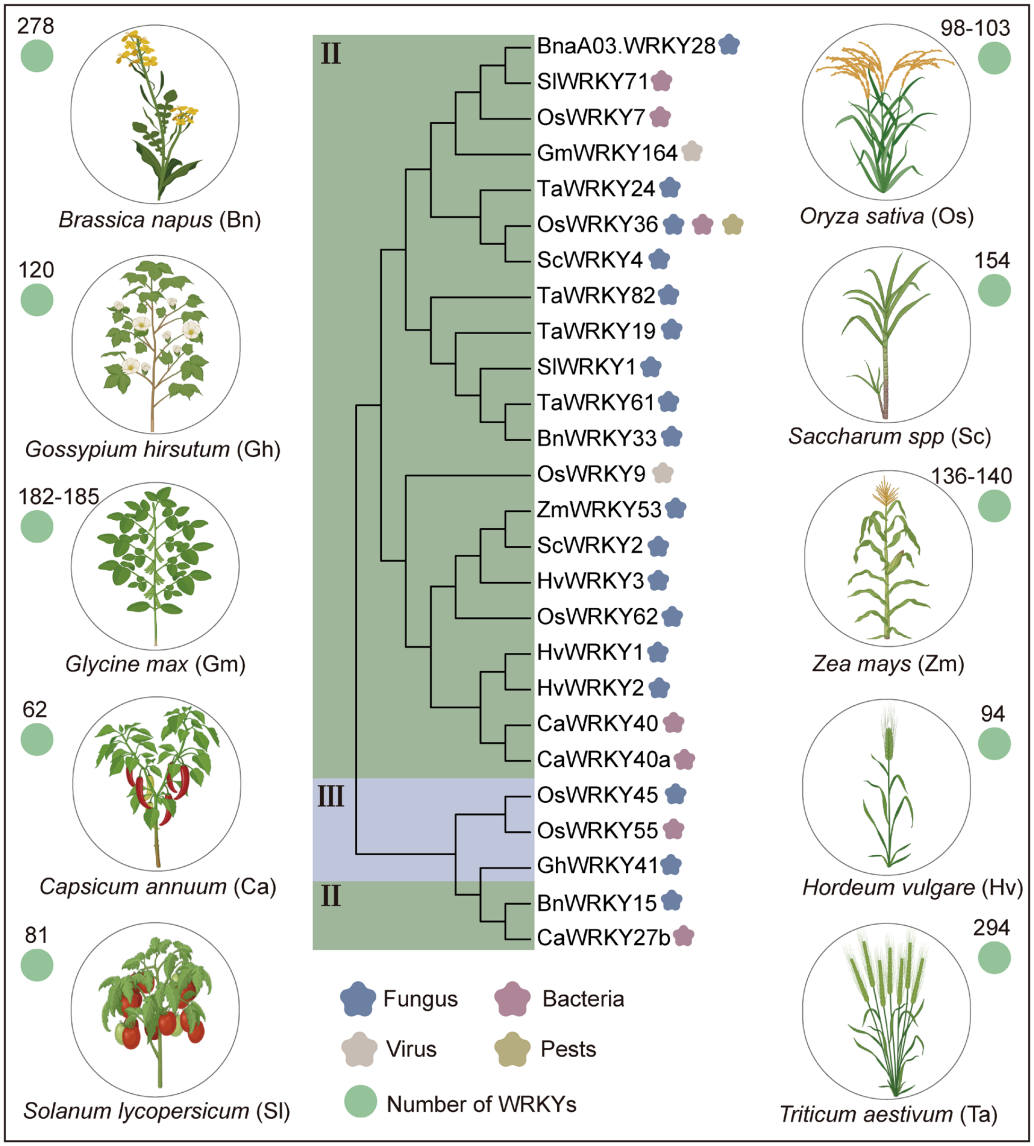
WRKY gene family in major crops. The phylogenetic tree was constructed using the neighbour‐joining method and illustrates representative WRKY transcription factors associated with various biotic stress responses across 12 major crop species. Two major clades (II and III) are distinguished by different background colours to indicate phylogenetic groupings. Coloured petal‐shaped hexagons represent the types of stress to which each *WRKY* gene responds. The blue colour represents responses to fungal diseases, purple denotes bacterial diseases, pink signifies viral diseases and gold highlights responses to pests. Green circles indicate the total number of *WRKY* genes identified in each species.

In plants, TFs play crucial roles in regulating gene expression and orchestrating diverse biological processes (Dhatterwal et al. 2024). Among them, the WRKY family is recognised as one of the earliest and most thoroughly characterised TF families within the plant kingdom (Rushton et al. 2010). Since the identification of a *WRKY* gene in sweet potato in 1994, members of this family have been characterised in a series of crops (Figure 2). Studies have progressively demonstrated the participation of WRKY TFs in sophisticated regulatory systems in various crops (Chen et al. 2017). In the model plant *A. thaliana* , 72 *WRKY* genes have been annotated, with 49 of them showing differential expression in response to *Pseudomonas syringae* infection or salicylic acid (SA) treatment (Dong et al. 2003). Notably, the promoters of these genes are significantly enriched with W‐box *cis*‐elements, which act as recognition sites for WRKY proteins. This observation confirms the existence of autoregulatory or feedforward mechanisms within the WRKY protein family.

Beyond *Arabidopsis*, comparative genomic analyses have uncovered substantial variation in the WRKY TF family across major crops, both in terms of gene number and functional specialisation (Figure 2). Gene counts range from 62 in pepper ( *Capsicum annuum* ) to 294 in wheat, reflecting striking interspecies differences in family size and composition (Yang, Huang, et al. 2025; Zheng et al. 2019). These genes are grouped into distinct subfamilies (I, IIa–IIe, III, and IV), which is consistent with patterns of lineage‐specific expansion and functional divergence (Yang, Huang, et al. 2025; Zheng et al. 2019). Such diversity is likely shaped by whole‐genome duplication events and the evolutionary pressures associated with distinct ecological contexts (Im et al. 2025; Inoue et al. 2013; Liu et al. 2025; Ramamoorthy et al. 2008; Ross et al. 2010; Shi et al. 2023; Wang, Zhou, et al. 2018; Zheng et al. 2024).

WRKY TFs have been extensively characterised in rice, particularly for their involvement in plant responses to biotic stresses (Im et al. 2025; Inoue et al. 2013; Liu et al. 2025; Pan et al. 2025; Ramamoorthy et al. 2008; Ross et al. 2010; Shi et al. 2023; Wang, Zhou, et al. 2018; Zheng et al. 2024). In other crops, functional studies are also rapidly emerging and contributing to a broader understanding of this gene family. For instance, in wheat, barley, and sugarcane, specific WRKY members such as *TaWRKY19*, *HvWRKY3,* and *ScWRKY2* are predominantly associated with resistance to fungal pathogens (Han et al. 2020; Wang et al. 2025; Wang, Fan, et al. 2022). On the other hand, *GmWRKY164* in soybean and *CaWRKY40* in pepper contribute to antiviral and antibacterial defence responses, respectively (Yang et al. 2022; Zhao et al. 2024). We observed that, while the immune‐related roles of many *WRKY* genes remain consistent across plant species, the development of unique regulatory modules specific to lineages highlights the significant functional diversity and adaptability within this gene family. Overall, these findings offer essential theoretical insights into stress‐response mechanisms and contribute to advancements in the genetic improvement of crops.

## 4 WRKY TFs Respond to Biotic Stress

Plants face a myriad of biotic stresses throughout their life cycle, including infections by fungi, bacteria, and viruses, as well as attacks from pests (Feng et al. 2024). These stresses significantly compromise plant growth and productivity, leading to substantial global losses in yield and quality. WRKY TFs serve as key regulators of plant defence mechanisms against biotic stresses (Jiang et al. 2017). Understanding WRKY‐mediated immune regulation not only deepens our understanding of plant immunity but also provides valuable targets and genetic resources for the development of disease‐ and pest‐resistant crop varieties. To enable comparison across different stress types, this review synthesises current evidence of WRKY‐mediated resistance within a cohesive framework that links genetically and functionally validated WRKYs to upstream immune signals, major signalling networks, and downstream defence responses. Using this framework, we summarise the roles of WRKY TFs in conferring resistance to fungal, bacterial, viral and pest‐associated stresses, emphasising conserved regulatory hubs and stress‐type‐specific features that hold translational significance for crop improvement, as detailed in Table 1.

**TABLE 1:** Representative WRKY transcription factors involved in biotic stress resistance.

Crop species	Genes	Gene id	Stress types	Disease resistance	Target genes	Protein interactions	References
Rice ( *Oryza sativa* )	*OsWRKY7*	LOC_Os05g46020	Bacterial pathogen: *Xanthomonas oryzae* pv. *oryzae* (*Xoo*)	Positive	—	—	Zheng et al. (2024)
	*OsWRKY9*	LOC_Os01g18584	Fungal pathogen: *Magnaporthe oryzae*; Bacterial pathogen: *Xoo*; Viral pathogen: rice stripe mosaic virus (RSMV)	Positive	—	—	Pan et al. (2025)
	*OsWRKY10*	LOC_Os01g09100	Bacterial pathogen: *Xoo*	Positive	*OsPR1a*, *OsWRKY47*	—	Choi et al. (2020)
	*OsWRKY31*	LOC_Os03g20550	Fungal pathogen: *M. oryzae*	Positive	—	OsMKK10, OsREIW1	Wang, Han, et al. (2023), Wang, Wang, et al. (2023)
			Fungal pathogen: *Ustilaginoidea virens*, *M. oryzae* , *Rhizoctonia solani*; Bacterial pathogen: *Xoo*	Positive	*OsAOC*	—	Duan et al. (2024 )
	*OsWRKY36*	LOC_Os04g46060	Fungal pathogen: *M. oryzae* ; Bacterial pathogen: *Xoo*; Pests: brown planthopper (BPH), white‐backed planthopper (WBPH), small brown planthopper (SBPH)	Negative	*OsPAL6*, *OsPAL1/6*, *IPA1*, *MOC2*	—	Liu et al. (2025)
	*OsWRKY53*	LOC_Os05g27730	Bacterial pathogen: *Xoo*	Positive	*OsMYB63*	—	Xie et al. (2021)
			Fungal pathogen: *M. oryzae*	Positive	*PBZ1*	OsDLA	Meng et al. (2024)
	*OsWRKY62*	LOC_Os09g25070	Fungal pathogen: *M. oryzae*	Positive	—	ANIP1, AvrPi9, Pi9	Shi et al. (2023)
	*OsWRKY67*	LOC_Os05g09020	Fungal pathogen: *U. virens*	Positive	*OsNOMT*	OsMPK6	Ma, Wei, et al. (2025)
	*OsWRKY82*	LOC_Os08g17400	Bacterial pathogen: *Xoo*	Positive	—		Yuan et al. (2024)
Maize ( *Zea mays* )	*ZmWRKY53*	Zm00001d020492	Fungal pathogen: *Sporisorium reilianum*	Negative	—	ZmSnRK1α2	Zhang et al. (2024)
Wheat ( *Triticum aestivum* )	*TaWRKY19*	TraesCS2D02G190500	Fungal pathogen: *Puccinia striiformis* f. sp. *tritici* (*Pst*)	Negative	*TaNOX10*	—	Wang et al. (2024)
	*TaWRKY50*	TraesCS3B01G199000.1	Viral pathogen: Chinese wheat mosaic virus (CWMV)	Positive	*TaSAPK7*, *NbSRK*	—	Guo et al. (2024)
Barley ( *Hordeum vulgare* )	*HvWRKY3*	AK359706.1	Fungal pathogen: *Blumeria graminis* f. sp. *hordei* (*Bgh*)	Negative	—	SnRK1	Han et al. (2020)
Sugarcane (*Saccharum spp*)	*ScWRKY2*	MK629766	Fungal pathogen: *S. scitamineum*	Negative	*ScLRR‐RLK*	ScPsbP	Wang et al. (2025)
Soybean ( *Glycine max* )	*GmWRKY164*	Glyma.17G224800	Viral pathogen: Soybean mosaic virus (SMV)	Positive	*GmGSL7c*	—	Zhao et al. (2024)
Cotton ( *Gossypium hirsutum* )	*GhWRKY40a*	—	Fungal pathogen: *Verticillium dahliae*	Positive	*GhERF1b*, *GhABF2*	GhMPK9, GhRAF39_1	Mi et al. (2024)
	*GhWRKY41*	Gh_A08G2417	Fungal pathogen: *V. dahliae*	Positive	*GhC4H*, *Gh4CL*	*GhWRKY41*	Xiao et al. (2023)
Tomato ( *Solanum lycopersicum* )	*SlWRKY30*	Solyc10g009550	Bacterial pathogen: *Ralstonia solanacearum*	Positive	*SlPR‐STH2a/b/c/d*	SlWRKY52/59/80/81	Dang et al. (2023)
	*SlWRKY30IIc*	Solyc07g056280	Pests: *Meloidogyne incognita*	Positive	*SlJAZ3*, *SlJAZ7*, *SlJAZ9*, *SlJAZ11*	SlVQ15	Huang et al. (2025)
	*SlWRKY71*	Solyc02g071130	Bacterial pathogen: *Pseudomonas syringae* pv. *tomato* (*Pst*) DC3000	Positive	*SlDCD1*	—	Zhao et al. (2025)
	*SlWRKY75*	Solyc05g015850	Bacterial pathogen: *Pst* DC3000	Positive	*SlGH3.3*	SlVQ16	Yang et al. (2024)
Pepper ( *Capsicum annuum* )	*CaWRKY27b*	XP_016566888	Bacterial pathogen: *R. solanacearum*	Positive	—	CaCDPK29, CaWRKY40	Yang et al. (2022)
	*CaWRKY01*	—	Oomycete pathogen: *Phytophthora capsici*	Positive	*CaPR1*, *CaPR4*, *CaPR4b*, *CaPR*	CaWRKY08	Cheng et al. (2024)
Tobacco (*Nicotiana benthamiana*)	*NbWRKY40*	Niben101Scf04944g05002.1	Viral pathogen: Tomato mosaic virus (ToMV)	Positive	*NbICS1*	—	Jiang et al. (2020)
	*NbWRKY45*	NbL08g18360.1	Pests: *Bemisia tabaci*	Positive	*NbC4H*, *Nb4CL*	—	Ji et al. (2024)
	*NbWRKY81*	NbL14g08830.1	Pests: *Bemisia tabaci*	Positive	*NbCCR*	—	Ji et al. (2024)
Grapevine ( *Vitis vinifera* )	*VqWRKY56*	—	Fungal pathogen: *Podosphaera leucotricha*	Positive	*VvCHS3*, *VvLAR1*, *VvANR*	VqbZIPC22	Wang, Wang, et al. (2023)
Apple ( *Malus domestica* )	*MdWRKY100*	MDP0000514115	Fungal pathogen: *Colletotrichum fructicola*	Positive	*MdWRKY17*, *MdPAL1*, *MdRPM1*	MdVQ37	Dong et al. (2024)

### 4.1 Fungal Stress

**FIGURE 3:**
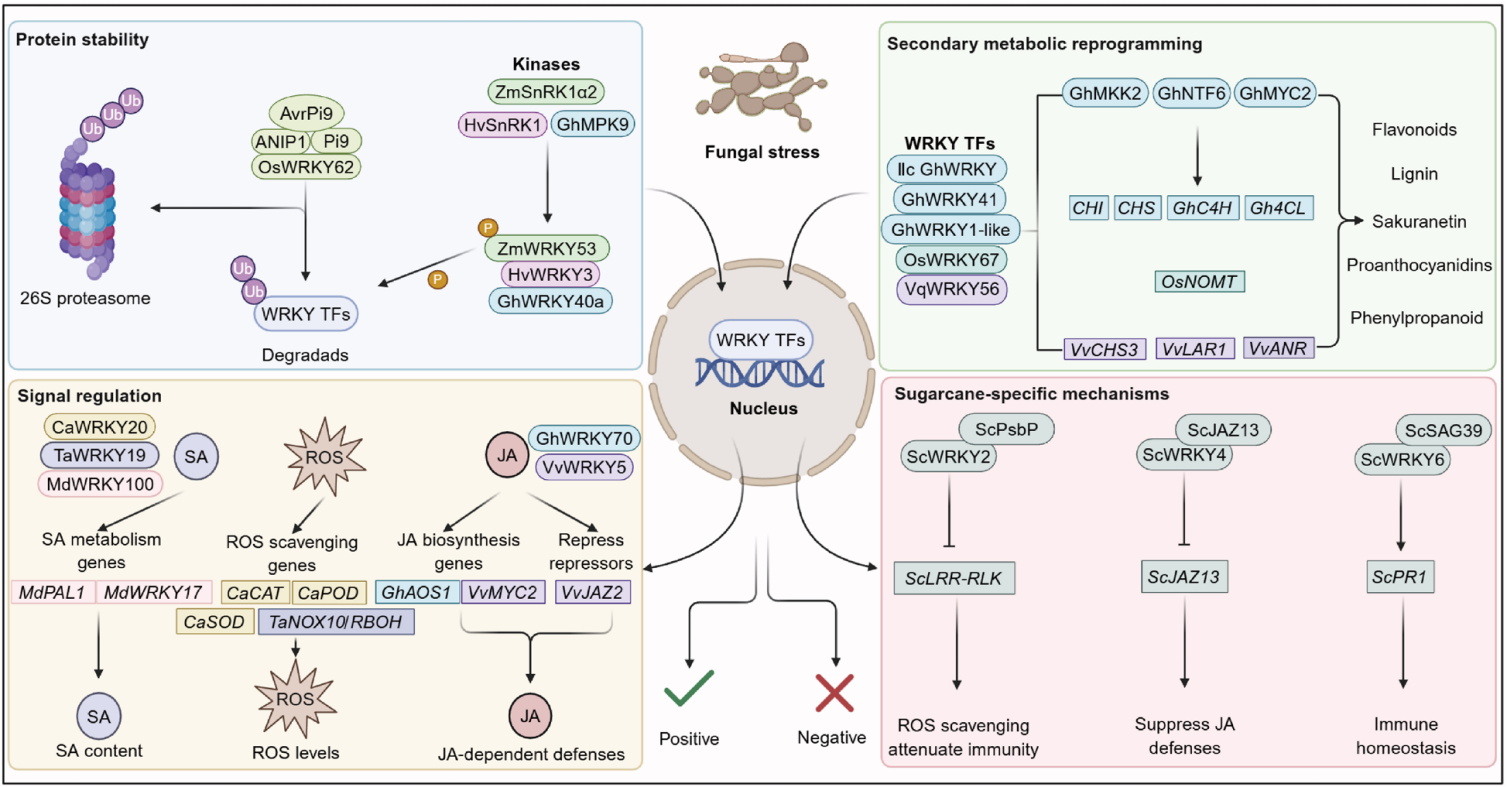
Roles of WRKY transcription factors in crop responses to fungal stress. This figure illustrates the regulatory mechanisms of WRKY transcription factors and their associated factors in plant resistance to fungal diseases, primarily focusing on three key aspects: Protein stability, secondary metabolic reprogramming, and signalling regulation. The upper left portion of the figure illustrates the role of WRKY protein stability in immune responses. The upper right portion highlights secondary metabolic reprogramming, particularly the functions of genes such as *GhWRKY1‐like*, *VqWRKY56*, *OsWRKY67,* and *GhWRK41* in regulating genes associated with antifungal metabolite synthesis. Furthermore, the figure elucidates the regulatory mechanisms of WRKY transcription factors at the signalling regulation level, demonstrating the interactions of genes *MdPAL1*, *MdWRKY17*, *CaCAT*, *CaPOD*, *CaSOD*, *TaNOX/RBOH*, *GhAOS1*, *VvMYC2,* and *VvJAZ2* with signalling pathways involving ROS, SA and JA. Arrows represent activation, while T‐bar lines indicate suppression. A brown circle labelled P indicates phosphorylation. A purple circle labelled Ub indicates ubiquitination. A rounded rectangle represents proteins, and a right‐angled rectangle represents genes. Different coloured ovals represent degraded WRKY. AvrPi9, rice blast pathogen‐secreted effector; Pi9, blast resistance gene; ROS, reactive oxygen species; SA, salicylic acid; JA, jasmonic acid.

Fungal pathogens are among the most extensively studied classes of biotic stress and have served as a main framework for understanding WRKY‐mediated immune responses. In this context, WRKY TFs serve as central regulators of plant responses to fungal diseases (Figure 3), orchestrating immune signalling and executing diverse functions in defence.

Protein stability plays a critical role in WRKY function. Its regulation involves degradation through the ubiquitin‐proteasome and phosphorylation by kinases. For instance, during ETI responses against *Magnaporthe oryzae* in rice, the resistance protein Pi9 recognises the effector AvrPi9, initiating immune signalling (Shi et al. 2023). OsWRKY62 serves as a crucial regulator in this pathway, with its protein stability modulated by the ubiquitin‐like domain‐containing protein (UDP) AvrPi9‐interacting protein 1 (ANIP1). In the absence of Pi9, ANIP1 facilitates the degradation of OsWRKY62 via the 26S proteasome, consequently suppressing the expression of defence genes and basal immunity—a process further enhanced by AvrPi9. In *Pi9*‐expressing plants, ANIP1, OsWRKY62, and Pi9 form a ternary complex that keeps Pi9 in an inactive state. The recognition of AvrPi9 releases Pi9 from this complex, thereby triggering ETI. In maize, the SnRK1 family kinase ZmSnRK1α2 phosphorylates ZmWRKY53, promoting its degradation (Zhang et al. 2024). This process inhibits the expression of genes related to transmembrane transport, glycolysis, and cell wall degradation (*ZmSWEETs*, *ZmPIP2;6*, *ZmHAK5*, *ZmPFKs*, *ZmPKs,* and *ZmXTHs*), thereby limiting the acquisition of host nutrition by *Sporisorium reilianum*. In barley, the protein kinase HvSnRK1 works similarly by attaching to and phosphorylating HvWRKY3, which promotes its degradation (Han et al. 2020). This reduces the haustorial and microcolony indices of *Blumeria graminis* f. sp. *hordei*, improving resistance to powdery mildew and underlining the conservation of the SnRK1‐WRKY signalling system in plant defence mechanisms. In cotton, the Raf‐like kinase GhRAF39_1 functions as an intermediate MAPK substrate that enables GhMPK9‐dependent phosphorylation of GhWRKY40a (Mi et al. 2024). When GhWRKY40a is activated, it promotes *GhERF1b* and downstream defence genes, strengthening resistance to Verticillium wilt. This is evident from the contrasting disease indices and vascular symptoms seen in plants with overexpressed *GhMPK9* compared to those where *GhMPK9*, *GhRAF39_1*, or *GhWRKY40a* are suppressed.

Secondary metabolic reprogramming constitutes a fundamental strategy by which WRKY TFs orchestrate plant disease resistance across multiple species. In cotton, Group IIc WRKY TFs directly bind to and activate the promoter of *GhMKK2*, a key component of the MAPK pathway, initiating the GhMKK2‐GhNTF6‐GhMYC2 cascade signalling pathway (Wang, Guo, et al. 2022). Consistently, virus‐induced gene silencing of Group IIc *WRKYs* or *GhMKK2* in cotton leads to more severe disease symptoms and significantly higher disease indices after *Fusarium oxysporum* infection, showing that they contribute positively to cotton's defence mechanisms. This activation upregulates the expression of flavonoid biosynthesis‐related genes, such as *chalcone isomerase* (*CHI*) and *chalcone synthase* (*CHS*), thereby improving plant resistance to *F. oxysporum* (Wang, Guo, et al. 2022). Concurrently, GhWRKY41 reinforces defence signalling via a positive feedback mechanism that upregulates phenylpropanoid pathway genes *cinnamate‐4‐hydroxylase* (*GhC4H*) and *4‐coumarate:CoA ligase* (*Gh4CL*), promoting lignin and flavonoid accumulation and enhancing resistance to *Verticillium dahliae* (Xiao et al. 2023). Similarly, the GhWRKY1‐like TF strengthens cell wall structure and effectively inhibits *V. dahliae* infection by targeting the lignin biosynthesis pathway, particularly promoting the biosynthesis of syringyl‐type monolignols (S monomers) (Hu et al. 2021). In rice, OsWRKY67 undergoes phosphorylation by OsMPK6, and thus induces the expression of *naringenin 7‐O‐methyltransferase* (*OsNOMT*) (Ma, Wei, et al. 2025). This process leads to the accumulation of the flavonoid phytoalexin sakuranetin, which directly inhibits the hyphal growth and proliferation of *Ustilaginoidea virens*, significantly reducing the incidence of false smut. In grapevine ( *Vitis vinifera* ), VqWRKY56 interacts with the basic leucine zipper (bZIP) TF VqbZIPC22 to co‐regulate the transcription of flavonoid biosynthetic genes, including *VvCHS3*, *VvLAR1,* and *VvANR*, resulting in proanthocyanidin accumulation that strengthens resistance to powdery mildew (Wang, Wang, et al. 2023). The aforementioned studies indicate that WRKY TFs enhance resistance by promoting secondary metabolite synthesis. However, the underlying mechanism of metabolite production in plant defence requires further investigation.

When WRKY TFs work alongside signalling pathways, plants gain a more finely adjusted immune response system. In the SA signalling pathway, WRKY proteins typically regulate disease resistance by coordinating the expression of genes involved in SA metabolism and ROS scavenging. CaWRKY20 may indirectly suppress the expression of SA‐related defence genes (*CaPR1*, *CaPR10,* and *CaSAR8.2*) and reactive oxygen species (ROS)‐scavenging related genes (*CaCAT*, *CaPOD,* and *CaSOD*), potentially weakening pepper resistance to *Colletotrichum scovillei* (Li et al. 2025). This leads to a reduction in ROS levels and an accumulation of SA, ultimately enhancing disease resistance in tomato leaves. In wheat, TaWRKY19 provides another example of WRKY‐mediated ROS regulation: as a *Puccinia striiformis* f. sp. *tritici* (*Pst*)‐inducible transcriptional repressor of *TaNOX10*, TaWRKY19 directly suppresses the TaNOX10/RBOH‐dependent ROS burst during infection by *Pst*, thereby attenuating basal immunity and increasing susceptibility to stripe rust (Wang, Fan, et al. 2022). Conversely, loss of *TaWRKY19* relieves this repression, enhances the ROS burst and restricts pathogen growth, conferring resistance even to highly virulent *Pst* races. MdWRKY100 binds to W‐box elements in the promoters of *MdWRKY17*, *MdPAL1,* and *MdRPM1* (Dong et al. 2024). It represses the SA degradation‐related gene *MdWRKY17* while activating the SA biosynthesis gene *MdPAL1* and the resistance gene *MdRPM1*. This increases SA content and enhances MdRPM1‐mediated resistance. MdVQ37 interacts with MdWRKY100 and inhibits its transactivation activity, thereby reducing SA content and *MdRPM1* expression and weakening resistance to glomerella leaf spot. In the jasmonic acid (JA) signalling pathway, *GhWRKY70* in cotton positively regulates resistance to Verticillium wilt by activating the expression of *GhAOS1*, a rate‐limiting enzyme in JA biosynthesis (Zhang, Dong, et al. 2023). In grapevine, *VvWRKY5* enhances resistance to white rot through coordinated regulation of the JA signalling pathway. It binds to the promoters of *VvJAZ2* and *VvMYC2*, repressing the negative regulator VvJAZ2 while activating the positive regulator VvMYC2 (Zhang, Jiang, et al. 2023). Together, these actions promote JA accumulation and JA‐dependent defence responses, thereby strengthening grapevine resistance to white rot.

Given the broad role of WRKY TFs in crop disease defence, we investigated their functions in sugarcane, a key tropical crop that is vulnerable to smut disease (*Sporisorium scitamineum*) (Wang et al. 2025, 2020, 2024; Wang, Liu, et al. 2018; Zang et al. 2025). Our group demonstrates that ScWRKY2 functions as a negative regulator of plant defence by repressing the transcription of the immune‐related gene *ScLRR‐RLK* through direct binding to the W‐box element in its promoter (Wang et al. 2025). Concurrently, ScWRKY2 interacts with the chloroplast‐associated protein ScPsbP, which induces the expression of ROS‐scavenging genes, thereby helping to maintain ROS homeostasis and ultimately attenuating the immune response. Additionally, ScWRKY4 negatively regulates pathogen responses probably through its potential interaction with ScJAZ13, thus suppressing JA‐mediated defence mechanisms (Wang et al. 2024). Building on this framework, Zang et al. (2025) identified ScWRKY6 positively regulates antifungal immunity by activating *ScPR1* and upregulating immune‐ and secondary metabolism‐related pathways. ScWRKY6 forms a tunable regulatory module with the endoplasmic reticulum‐associated cysteine protease ScSAG39, which limits its nuclear accumulation and interferes with W‐box binding. This interaction dampens *ScPR1* activation and contributes to immune homeostasis. These findings underscore the WRKY‐mediated transcriptional and protein‐level regulation in sugarcane immunity and lay a foundation for future research and breeding efforts.

### 4.2 Bacterial Stress

**FIGURE 4:**
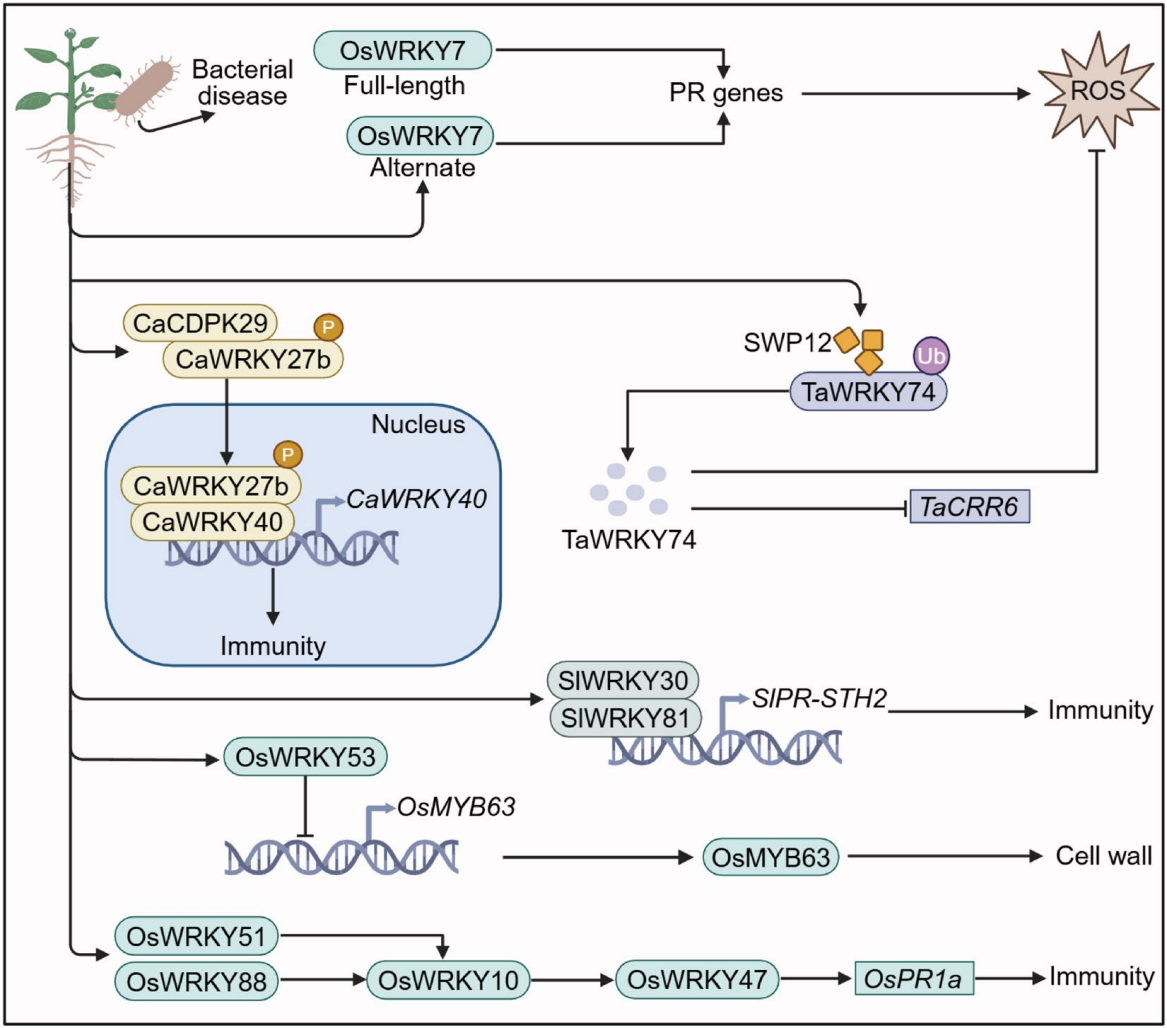
The roles of WRKY transcription factors in crop defence against bacterial stress. This figure illustrates the roles of WRKY transcription factors in regulating plant defence against bacterial infection via immune signalling, ROS accumulation, and cell wall biosynthesis. After bacterial infection, OsWRKY7 regulates the expression of PR genes through both full‐length and alternatively spliced forms, thereby inducing the generation of ROS and activating immune responses. CaWRKY27b interacts with CaCDPK29, translocates into the nucleus and promotes the transcription of CaWRKY40, thereby regulating immune‐related genes. TaWRKY74 cooperates with the SWP12 protein to modulate its ubiquitination, further regulating the expression of TaCRR6 and contributing to immune responses. Additionally, OsWRKY53 regulates OsMYB63, impacting cell wall synthesis and enhancing plant immune defence. Other WRKY transcription factors, such as OsWRKY51 and OsWRKY88, are involved in regulating the immune‐related gene *OsPR1a*, thereby further strengthening the plant's resistance. Arrows represent activation, while T‐bar lines indicate suppression. The rounded rectangle represents proteins, and the right‐angled rectangle represents genes. A brown circle labelled P indicates phosphorylation. A purple circle labelled Ub indicates ubiquitination. Different coloured ovals represent degraded WRKY. SWP12, a wheat blue dwarf phytoplasma effector. *PR* genes, pathogenesis‐related gene; ROS, reactive oxygen species.

In addition to their crucial roles in antifungal immunity, WRKY TFs are also extensively involved in immune pathways against bacterial infections. Many WRKYs function as central nodes linking early immune signals to downstream defence responses, thereby enabling plants to rapidly adjust transcriptional programmes, cell wall architecture and hormone homeostasis in response to bacterial attack. Below, we highlight representative WRKY‐centred regulatory modules that contribute to bacterial disease resistance in rice, tomato, pepper and other crops (Figure 4).

Functional diversity of WRKYs in antibacterial immunity is manifested at multiple regulatory levels, including protein isoform generation and post‐translational modification. In rice, OsWRKY7 illustrates how a single WRKY locus can produce distinct immune outputs (Zheng et al. 2024). Alternative translation initiation generates two protein isoforms, a full‐length isoform that is induced upon pathogen invasion and a truncated isoform that is constitutively expressed. Both isoforms retain transcription factor activity and jointly contribute to resistance against the bacterial blight pathogen *Xanthomonas oryzae* pv. *oryzae* (*Xoo*). This is evidenced by reduced lesion lengths and bacterial growth in lines with enhanced OsWRKY7 activity compared with wild‐type (WT) plants, underscoring the importance of WRKY dosage and protein diversity in fine‐tuning antibacterial defences. Post‐translational regulation provides an additional layer of control. In pepper, CaWRKY27b is phosphorylated by the Ca^2+^‐dependent protein kinase CaCDPK29 in a Ca^2+^‐dependent manner and subsequently relocalizes from the cytoplasm to the nucleus, where it activates transcription of *CaWRKY40* (Yang et al. 2022). This CaCDPK29‐CaWRKY27b‐CaWRKY40 module positively modulates resistance to bacterial wilt caused by *Ralstonia solanacearum* , illustrating how calcium‐dependent kinase cascades channel early signalling events into WRKY‐mediated transcriptional reprogramming.

WRKY TFs also impinge on structural defences by reprogramming cell‐wall biosynthesis. In rice, OsWRKY53 represses the expression of *OsMYB63*, a key regulator of secondary cell wall cellulose biosynthesis (Xie et al. 2021). This repression down‐regulates cellulose synthase genes (*OsCesA4/7/9*), compromises cell wall integrity, and increases susceptibility to *Xoo*. Conversely, silencing *OsWRKY53* or overexpressing *OsMYB63* promotes cellulose deposition and reinforces the cell wall barrier against bacterial invasion. In addition to its role in cell‐wall‐associated defence, nuclear OsWRKY53 activates brassinosteroid (BR)‐responsive genes and directly induces transcription of the BR gene *PBZ1*, thereby enhancing rice resistance to blast disease, highlighting that individual WRKYs can integrate structural and hormonal layers of immunity.

A second major dimension of WRKY‐mediated antibacterial defence lies in the coupling of immune responses with hormone and redox signalling. In tomato, SlWRKY71 functions as a *Pseudomonas syringae* pv. tomato (*Pst*) DC3000‐inducible transcriptional activator that directly binds W‐box elements in the promoter of the H_2_S‐synthesising gene *SlDCD1* and promotes its expression (Zhao et al. 2025). The resulting increase in H_2_S levels enhances SA accumulation, modulates reactive oxygen species (ROS) homeostasis and up‐regulates defence marker genes such as *SlPR1* and *SlNPR1*. Correspondingly, *SlWRKY71*‐overexpression plants develop milder bacterial speck symptoms and sustain lower *Pst* DC3000 titres than WT plants, whereas *Slwrky71* mutants are more susceptible. Another tomato module, SlVQ16‐SlWRKY75‐SlGH3.3, exemplifies how WRKYs fine‐tune auxin‐SA crosstalk during bacterial infection (Yang et al. 2024). SlWRKY75 cooperates with the VQ‐motif protein SlVQ16 to directly activate the IAA‐Asp synthetase gene *SlGH3.3*, driving the conversion of free IAA into IAA‐Asp. This shift in auxin homeostasis attenuates auxin signalling and expansin gene expression, enhances the expression of SA‐dependent defence genes *SlPR1* and *SlPR1B*, and ultimately promotes resistance to *Pst.* DC3000.

In rice, OsWRKY82 acts downstream of the growth‐regulating factor OsGRF6 to coordinate JA‐associated defence against *Xoo* (Yuan et al. 2024). OsGRF6 binds CGC(G)A(C)G(A) motifs in the *OsWRKY82* promoter and activates its transcription in response to *Xoo* infection and JA treatment. Nuclear OsWRKY82 then functions as a transcriptional activator that up‐regulates defence marker genes (*PR1b*, *PR4*, *PR5* and *PR10*), promotes H_2_O_2_ accumulation and co‐regulates JA‐related genes including *AOS2*, *AOS3*, *LOX6* and *LOX9*, thereby strengthening JA‐associated antibacterial defence without markedly affecting grain yield. Together, these examples illustrate that WRKY‐centred modules are key nodes at which SA, JA, auxin and gasotransmitter pathways converge to shape the amplitude and duration of antibacterial immunity.

Finally, WRKY TFs further expand their regulatory capacity through combinatorial interactions and transcriptional cascades. In tomato, SlWRKY30 physically interacts with SlWRKY81 to activate the PR gene *SlPR‐STH2*, conferring resistance to *R. solanacearum* (Dang et al. 2023). In rice, OsWRKY10 and OsWRKY88 are integrated into the OsWRKY51‐OsWRKY10‐OsWRKY47 and OsWRKY88‐OsWRKY10‐OsWRKY47 transcriptional cascades, which cooperatively promote the expression of basal immunity genes such as *OsPR1a* (Choi et al. 2020). These combinatorial and hierarchical setups enable WRKYs to create adaptable regulatory units that can be modified to address various bacterial pathogens and specific tissue environments. Altogether, WRKY TFs act as flexible centres linking bacterial detection to transcriptional, structural, and hormonal aspects of immune control, offering plants a strong and flexible defence mechanism against bacterial infections.

### 4.3 Oomycete, Viral and Insect Pest Stresses

In addition to fungal and bacterial diseases, WRKY TFs also regulate plant responses to oomycete and viral infections as well as insect pest attack. In pepper, two nuclear‐localised WRKY TFs, CaWRKY01 and CaWRKY08, are capable of binding to the W‐box elements in the promoters of *PR* genes and directly activating their transcription, thereby enhancing resistance to *Phytophthora capsici* (Cheng et al. 2024). Although both factors regulate the same target genes, no protein–protein interactions (PPIs) or co‐regulatory relationship was detected between them, suggesting that they function relatively independently in the immune response.

Within the context of antiviral immunity, WRKY TFs show contrasting roles: some contribute to fortifying host defences, while others are co‐opted by viruses to encourage infection, resembling their supportive and detrimental roles in fungal and bacterial stress responses. WRKY TFs represent a fundamental regulatory component in plant responses to viral diseases, playing a pivotal role in the intricate network of interactions between plants and viruses. Different WRKY family members actively contribute to establishing the plant's defence mechanisms by targeting specific downstream pathways; conversely, they may be exploited by viruses as auxiliary factors for infection, demonstrating functional duality. Nuclear‐localised WRKY TFs primarily regulate callose deposition at plasmodesmata, a conserved mechanism of disease resistance, by binding to W‐box *cis*‐acting elements in the promoters of target genes. In tobacco, NbWRKY40 interacts with the W‐box element on the *NbICS1* promoter, which activates SA biosynthesis and upregulates the expression of SA‐signal genes *PR1b* and *PR2* (Jiang et al. 2020). The resultant increase in SA levels, alongside the activation of CalS1, facilitates callose deposition, thereby specifically obstructing the intercellular and systemic spread of Tomato mosaic virus (ToMV) without hindering viral replication. *NbWRKY40*‐silenced plants display reduced callose accumulation, faster viral movement, and more severe mosaic symptoms than WT plants, linking WRKY‐dependent structural reinforcement to higher antiviral efficiency. This mechanism is similarly conserved in soybean resistance to Soybean mosaic virus (SMV), where GmWRKY164 enhances the transcriptional activity of *GmGSL7c* by binding to the W‐box element on its promoter, facilitating callose deposition at plasmodesmata and restricting intercellular viral spread (Zhao et al. 2024). Silencing *GmWRKY164* significantly decreases callose deposition, accelerates virus movement and results in higher virus titers and more severe disease symptoms compared to WT plants. This provides crop‐specific evidence that WRKY‐regulated callose barriers constitute a general antiviral strategy.

In addition to establishing physical barriers to defend against viruses, WRKY TFs also play a pivotal role in regulating RNA interference (RNAi), a fundamental antiviral immune pathway in plants. Following viral infection, WRKY1 is upregulated and binds to the *NbWHIRLY1* (*NbWhy1*) promoter, which inhibits *NbWhy1* expression (Sun et al. 2023). This action alleviates the negative regulation imposed by *NbWhy1* on the RNAi defence pathway, thereby enhancing the plant's antiviral immune response. The overexpression of *NbWhy1* facilitates viral proliferation, whereas silencing *NbWhy1* increases plant resistance, clearly underscoring the critical role of the WRKY1‐*NbWhy1* signalling cascade in modulating plant immune responses.

Unlike the WRKY members that function through active disease resistance mechanisms, some WRKY TFs can be recruited by viruses as co‐regulatory factors to promote viral infection. TaWRKY50 plays a crucial role in wheat infection by the Chinese wheat mosaic virus (CWMV) (Guo et al. 2024). As a transcriptional activator, it enhances the expression of *TaSAPK7* and *NbSRK* by binding to their promoters. During CWMV infection, TaSAPK7 and NbSRK kinases phosphorylate cysteine‐rich proteins (CRPs), and TaWRKY50 amplifies the phosphorylation of CRPs by activating the expression of these kinases while simultaneously inhibiting programmed cell death (PCD). This inhibition prevents the cell‐autonomous defence of the plant immune response. Silencing *TaWRKY50* results in reduced CRP phosphorylation levels and initiates PCD, thereby inhibiting CWMV replication and validating its function in promoting viral infection by regulating protein modification and cell death processes. This duality of function reflects the flexibility of the WRKY transcription factor family in plant‐virus interactions and reveals the complex interplay of offensive and defensive mechanisms in these interactions.

WRKY TFs play a key role in protecting plants from pests by orchestrating multilayered regulatory networks, including the activation of structural defence pathways such as lignin biosynthesis. Whitefly feeding triggers a notable increase in lignin production within *N. benthamiana* as a defensive response. Yet, when key lignin biosynthesis genes (*NbC4H*, *Nb4CL* and *NbCCR*) are silenced, this protective mechanism weakens. Consequently, whiteflies not only survive but also thrive, laying more eggs with greater success (Ji et al. 2024). Subsequent analyses revealed that NbWRKY45 and NbWRKY81 transcriptionally activate these lignin biosynthetic genes, thereby promoting lignification and enhancing insect resistance (Ji et al. 2024). Similarly, in rice, knockout of *OsWRKY36* results in increased lignin deposition and thickened sclerenchyma cells in the leaf sheath, conferring broad‐spectrum resistance to brown planthopper, white‐backed planthopper and grey planthopper (Liu et al. 2025). Moreover, WRKY TFs contribute to defence against insect herbivores by directly regulating structural defence genes and modulating hormone signalling pathways through PPIs. In sorghum, a genome‐wide association study identified *SbWRKY86* as a candidate gene for resistance to sugarcane aphid ( *Melanaphis sacchari* ) (Poosapati et al. 2022). Ectopic expression of *SbWRKY86* in tobacco and *Arabidopsis* significantly suppresses aphid population growth by enhancing callose deposition in cell walls and restricting phloem access. Furthermore, WRKY proteins frequently collaborate with VQ‐domain proteins to modulate hormone signalling. For instance, the tomato protein SlVQ15 physically interacts with SlWRKY30IIc to repress the expression of JA signalling repressors (*SlJAZ3*, *SlJAZ7*, *SlJAZ9* and *SlJAZ11*), thereby activating JA‐mediated systemic resistance against root‐knot nematodes (Huang et al. 2025).

**FIGURE 5:**
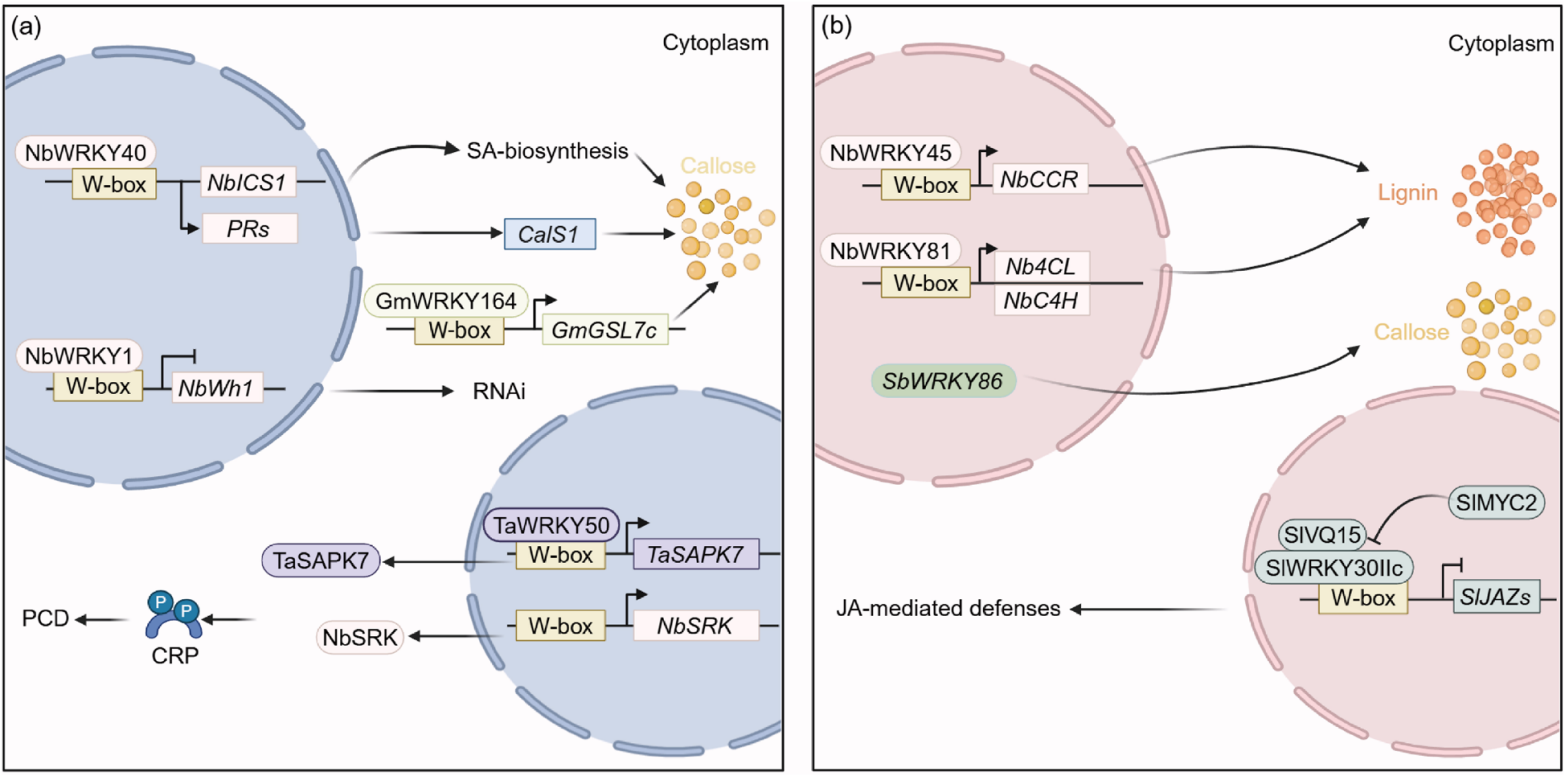
Functions of WRKY transcription factors in crop defence against viral infections (a) and pests (b). This figure illustrates the role of WRKY transcription factors in regulating plant defence mechanisms against viruses (a) and pests (b). During viral infection, *NbWRKY40* in tobacco promotes the expression of SA biosynthesis genes such as *ICS1* and SA signalling genes including *PRs* (*PR1b* and *PR2*), enhancing callose deposition and limiting viral movement. In soybean, *GmWRKY164* regulates the glucan synthase gene *GmGSL7c*, inhibiting soybean mosaic virus movement. WRKY TFs also regulate antiviral immunity through PCD and RNAi pathways. In wheat, *TaWRKY50* activates *TaSAPK7* and *NbSRK*, inhibiting viral RNA silencing suppressor CRP and enhancing PCD. NbWRKY1 represses *NbWh1* to enhance RNA silencing and resistance to geminiviruses. Additionally, NbWRKY45 and NbWRKY81 promote lignification by activating lignin biosynthesis genes (*NbC4H*, *Nb4CL*, and *NbCCR*), thereby enhancing insect resistance. In sorghum, *SbWRKY86* suppresses aphid growth by enhancing callose deposition, while SlVQ15 interacts with SlWRKY30IIc to activate JA‐mediated resistance against root‐knot nematodes. Arrows represent activation, while T‐bar lines indicate suppression. A rounded rectangle represents a protein, and a right‐angled rectangle represents a gene. CRP, cysteine‐rich protein; PCD, programmed cell death.

Taken together, these studies demonstrate that WRKY TFs regulate plant responses to stresses caused by oomycetes, viruses and insect pests by activating *PR* genes, fine‐tuning SA and JA signalling pathways, promoting the deposition of callose and lignin, and modulating RNAi and PCD. When considered alongside the fungal and bacterial cases discussed earlier, these findings support a model in which WRKY‐centred networks provide a shared mechanistic foundation for engineering broad‐spectrum and durable disease and pest resistance in crops (Figure 5).

## 5 WRKY TFs Mediate the Trade‐Off Between Defence and Growth

**FIGURE 6:**
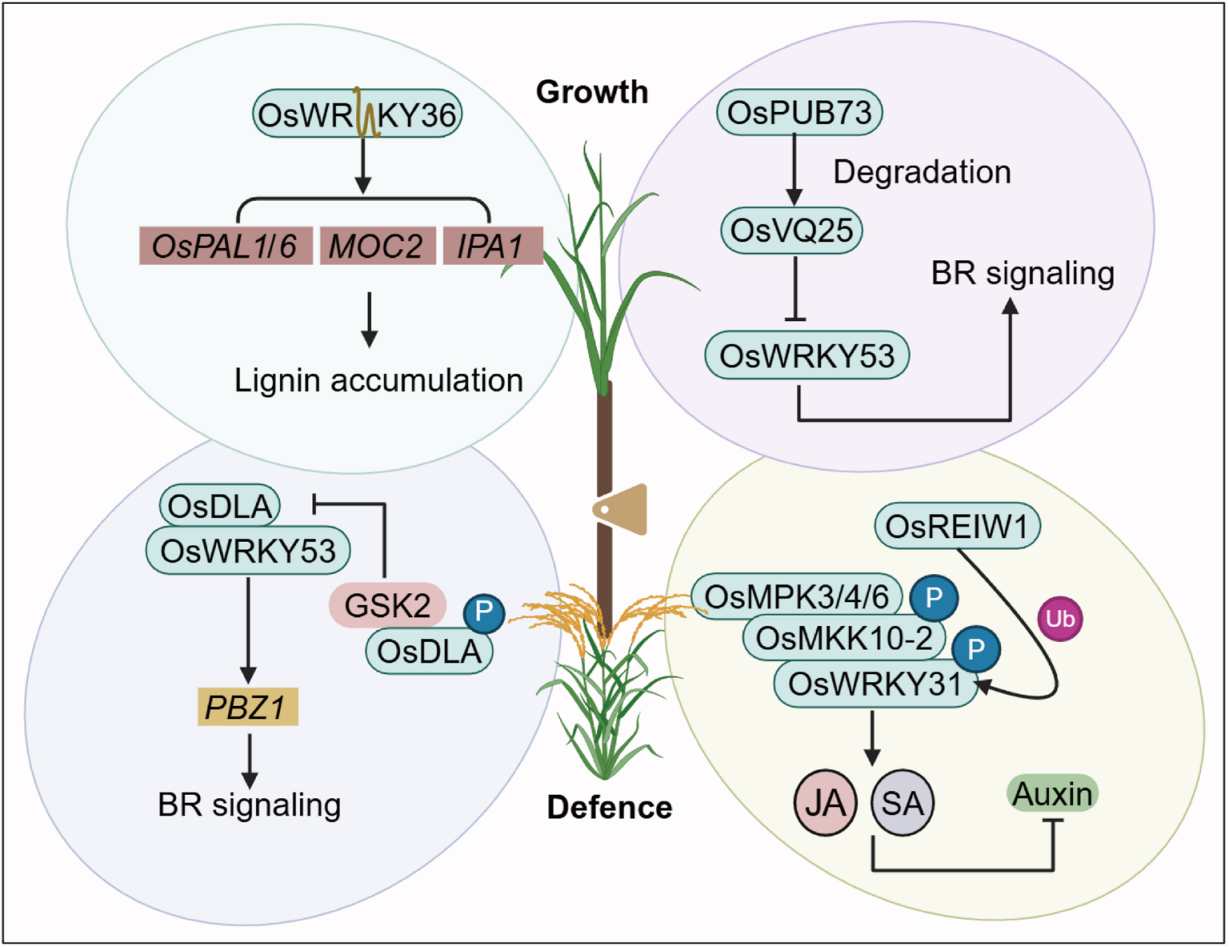
Regulation of defense and growth by WRKY transcription factors. This figure illustrates the molecular mechanisms by which multiple WRKY transcription factors coordinate growth and immune responses in rice. OsWRKY36 diminishes resistance to pathogens and pests by repressing the transcription of the lignin biosynthesis gene *PAL*. Conversely, its knockout leads to increased lignin accumulation, thereby enhancing resistance and improving yield. OsWRKY53 regulates the interplay between defence and growth hormone signalling through a regulatory module that includes the U‐box E3 ligase OsPUB73 and the VQ protein OsVQ25. Furthermore, OsWRKY53 interacts with the GRAS family transcription factor OsDLA, activating the expression of the blast resistance gene *PBZ1*. The BR signalling kinase GSK2 phosphorylates OsDLA, resulting in its degradation and modulating the stability of the OsWRKY53–OsDLA complex, thereby linking BR signalling to immune regulation. Additionally, OsWRKY31 participates in the MAPK cascade to enhance immune responses, with phosphorylation increasing its DNA‐binding affinity and promoting the expression of defence genes, thereby strengthening resistance to rice blast disease. Arrows represent activation, while T‐bar lines indicate suppression. The rounded rectangle represents proteins, and the right‐angled rectangle represents genes. A brown circle labelled P indicates phosphorylation. A purple circle labelled Ub indicates ubiquitination. BR, brassinosteroid; JA, jasmonic acid; SA, salicylic acid.

Plants face challenges in balancing defence responses and growth. WRKY TFs help connect these processes for optimal outcomes (Figure 6). In rice, *OsWRKY36* suppresses lignin biosynthesis by repressing the transcription of *Phenylalanine Ammonia Lyase* (*PAL*) gene (Liu et al. 2025). This repression reduces its resistance to rice blast, bacterial blight, and insect pests. Conversely, the knockout of *OsWRKY36* increases lignin accumulation and thickens the leaf sclerenchyma (Liu et al. 2025). Additionally, the loss of *OsWRKY36* derepresses the *Ideal Plant Architecture 1* (*IPA1*) and *MONOCULM2* (*MOC2*) genes, resulting in increased spikelet number per panicle and tiller number (Liu et al. 2025). These changes lead to higher grain yield and improved disease resistance, illustrating how OsWRKY36 balances defence and growth. OsWRKY53 is another key regulator that functions within a regulatory module comprising the U‐box E3 ligase OsPUB73 and the VQ‐motif protein OsVQ25 (Hao et al. 2022). To be specific, OsPUB73 promotes the proteasomal degradation of OsVQ25, thereby alleviating its repression of OsWRKY53 (Hao et al. 2022). This regulatory cascade modulates the expression of OsWRKY53 target genes involved in defence and BR signalling, enabling a dynamic balance between immune responses and plant growth (Hao et al. 2022). As discussed above in the context of WRKY‐mediated responses to fungal stress, in rice the ANIP1‐OsWRKY62‐Pi9 module coordinately regulates basal defence and Pi9‐mediated ETI against the rice blast fungus *M. oryzae* (Shi et al. 2023). ANIP1 promotes proteasome‐dependent degradation of OsWRKY62 in a Pi9‐dependent manner, thereby modulating blast resistance. In the Nipponbare background, *oswrky62* mutants exhibit increased grain length and hundred‐grain weight, with little or no effect on vegetative growth, whereas in the Nipponbare‐Pi9 background *oswrky62* mutants display a dwarf phenotype. These observations indicate that manipulation of OsWRKY62 can simultaneously alter disease resistance and growth‐related traits in a genotype‐dependent manner.

The integration of hormone signalling and immunity is further exemplified by the GSK2‐OsDLA‐OsWRKY53 module (Meng et al. 2024). OsDLA, a TF belonging to the GRAS family, interacts with OsWRKY53 to activate the expression of the blast‐resistance gene *PBZ1* (Meng et al. 2024). However, the BR‐signalling kinase GSK2 phosphorylates OsDLA, leading to its degradation (Meng et al. 2024). This post‐translational modification weakens the OsDLA‐OsWRKY53 complex, thereby linking brassinosteroid (BR) signalling to immune regulation and coordinating plant architecture with blast resistance (Meng et al. 2024). OsWRKY31, a multifunctional transcription factor in rice, plays a pivotal role in MAPK cascade‐driven immune responses (Wang, Han, et al. 2023). Functioning downstream of the OsMKK10‐2‐OsMPK3/4/6 module, OsWRKY31 undergoes direct phosphorylation, a modification that augments its DNA‐binding affinity and promotes the expression of defence‐related genes (Wang, Han, et al. 2023). This increases the accumulation of SA and JA, while simultaneously repressing auxin biosynthesis, thereby strengthening resistance to Magnaporthe oryzae at the expense of growth (Wang, Han, et al. 2023). Furthermore, the stability of OsWRKY31 is finely regulated by ubiquitination‐dependent degradation through the RING‐finger E3 ligase OsREIW1, highlighting a multilayered post‐translational regulatory mechanism (Wang, Han, et al. 2023). Collectively, these findings position OsWRKY31 as a central integrator of phosphorylation and ubiquitination cues in coordinating rice immunity and development.

Taken together, all these above studies underscore the pivotal role of WRKY TFs as integrators for developmental cues and immune responses under biotic stress. The multifaceted regulation of WRKYs, which includes but not limited to transcriptional control, PPIs, phosphorylation and ubiquitin‐mediated degradation, enables plants to fine‐tune the trade‐off between growth and defence in a context‐dependent manner. The regulatory flexibility is crucial for optimising plant fitness in dynamic environments. Moreover, the dual roles of WRKY TFs in modulating both architectural traits and pathogen resistance present promising opportunities for crop improvement strategies that preserve yield while enhancing stress resilience.

## 6 Concluding Remarks and Future Perspectives

WRKY TFs are plant‐specific proteins that play pivotal roles in mediating crop responses to biotic stresses (Rushton et al. 2012; Yang, Fang, et al. 2025). This review provides a comprehensive overview of recent advances in elucidating WRKY functions during biotic stress responses in crops. We summarise their structural features and classification, genome‐wide identification across major crop species, and their involvement in molecular pathways activated by fungal, bacterial, viral, and insect attacks. Additionally, we highlight their central regulatory roles in coordinating defence signalling with growth and developmental processes. Studies have shown that WRKY TFs are essential components of crop immunity, managing various hormone signalling processes, overseeing secondary metabolite production, guiding programmed cell death, and collaborating with other immune‐regulating proteins (Guo et al. 2024; Huang et al. 2025; Wang et al. 2024; Wang, Guo, et al. 2022; Xiao et al. 2023; Zhang et al. 2024). As studies advance, it is probable that new WRKY versions with unique structural designs and different roles will be discovered. These newly found types may not fit into current classification models or functional understandings, possibly resulting in the creation of new subcategories or even additional primary groups within the WRKY family.

Despite substantial progress, a comprehensive understanding of WRKY functions remains elusive. The WRKY family is large and diverse, characterised by considerable structural variation, functional redundancy and complex intra‐family interactions. Among those WRKY members, systematic dissection of functional divergence and regulatory coordination is still limited but urgently needed. Most current research has primarily focused on individual stress conditions; however, integrated analyses of WRKY‐mediated responses to simultaneous biotic stresses under field‐relevant conditions remain largely lacking. Furthermore, the potential ‘non‐classical’ roles of WRKY proteins, including their involvement in epigenetic regulation and RNA metabolism, warrant further investigation.

In terms of practical applications, WRKY TFs are key tools in molecular breeding efforts to achieve broad‐spectrum disease resistance in crops. Accumulating evidence proves that manipulating specific *WRKY* genes through genetic engineering approaches, such as overexpression or CRISPR‐Cas‐based editing, can effectively amplify plant defence responses, thereby improving resistance against diverse pathogenic taxa (Li et al. 2022; Liu et al. 2025; Pan et al. 2025; Rönspies et al. 2021; Zhou et al. 2024). To date, more and more scientists strive to explore strategies that leverage WRKY‐mediated regulatory networks to simultaneously optimise disease resistance and plant growth. For instance, the CRISPR‐Cas‐mediated knockout of *OsWRKY36* in rice has been shown to confer both broad‐spectrum disease resistance and satisfied yield‐associated traits (Liu et al. 2025). Similarly, the OsVQ25‐OsWRKY53 regulatory module exemplifies the role of WRKY proteins in modulating the balance between immunity and growth through precise PPIs (Hao et al. 2022). These insights collectively propose viable strategies for engineering elite crop varieties that integrate enhancements in both immunity and agronomic performance. Collectively, these findings provide strategic directions for engineering elite crop varieties with enhanced immunity and agronomic performance. To fully harness the disease‐resistance potential of *WRKY* genes, advanced technologies are being integrated into both experimental and computational frameworks. Artificial intelligence (AI)‐assisted CRISPR‐Cas target design facilitates precise and multiplexed genome editing, overcoming functional redundancy within the WRKY gene family and enhancing resistance durability. In parallel, generative AI approaches, including large language models and automated knowledge graphs, are utilised to identify WRKY‐related regulatory elements, gene networks, and signalling cascades from extensive literature and biological databases. These integrative strategies underscore the regulatory significance of WRKY TFs and present actionable solutions to expedite resistance‐focused crop genetic improvement.

**FIGURE 7:**
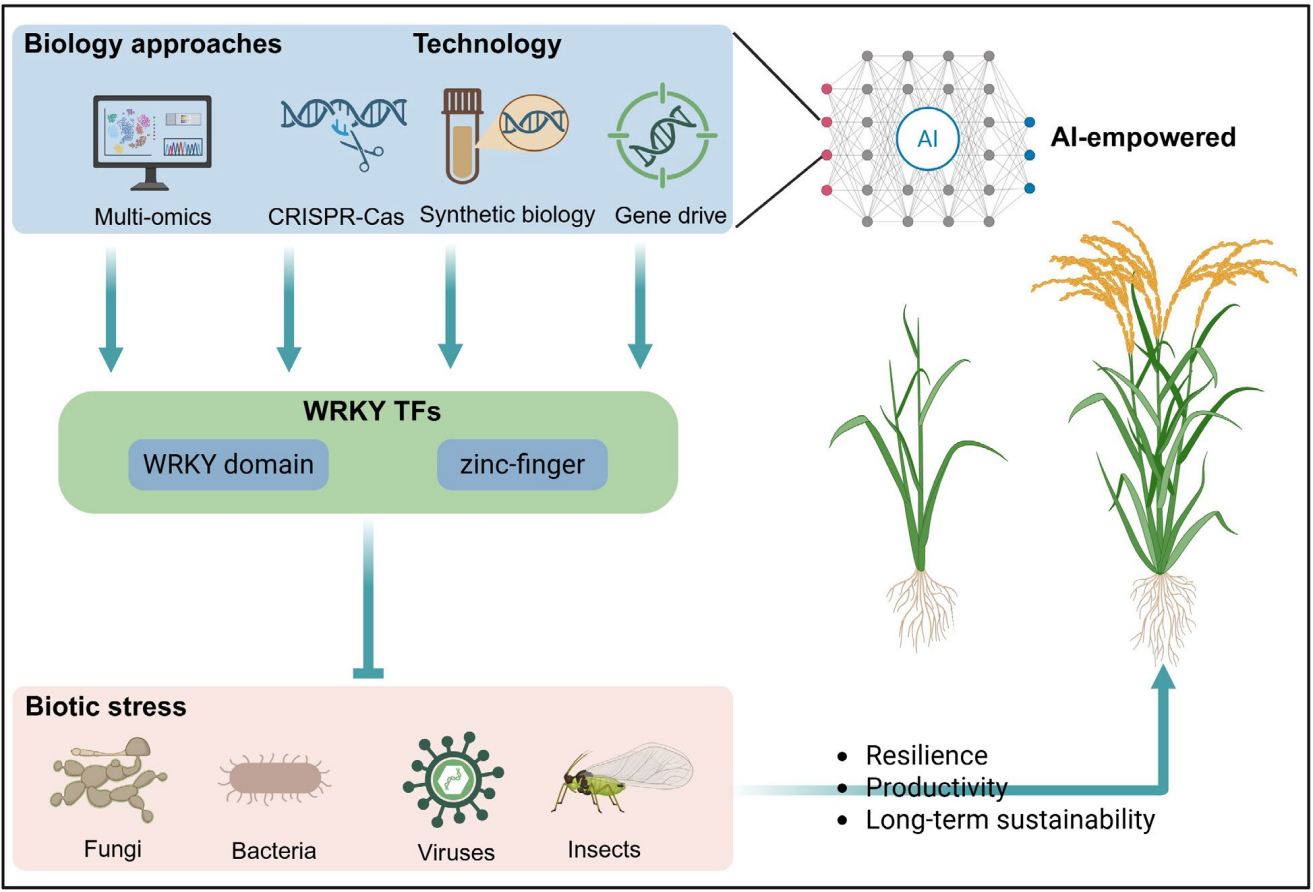
Potential uses of WRKY transcription factors in crop genetic improvement. This figure illustrates a conceptual framework for optimising crop performance under biotic stress through the application of WRKY transcription factors. By leveraging artificial intelligence (AI)‐empowered multi‐omics approaches alongside advanced technologies such as genome editing (CRISPR‐Cas), synthetic biology, and gene drive, scientists are able to pinpoint and accurately adjust specific WRKY transcription factors along with their regulatory systems. These approaches aim to boost plants' ability to withstand threats from fungi, bacteria, viruses, and insects, ultimately enhancing crop stress tolerance, increasing productivity, and supporting sustainable agriculture for the future.

Looking ahead, future research should prioritise the delineation of WRKY‐centred regulatory networks through systems biology approaches that integrate AI‐empowered multi‐omics analyses, including transcriptomics, proteomics, and epigenomics. The incorporation of emerging technologies, such as CRISPR‐Cas‐based genome editing, synthetic biology, and gene drive systems, alongside artificial intelligence, will facilitate the efficient and precise molecular engineering of broad‐spectrum disease resistance in crops. As our understanding continues to expand, the functional versatility and regulatory complexity of WRKY TFs are anticipated to extend beyond biotic stress responses, thus providing valuable insights to enhance crop resilience, productivity and long‐term sustainability in agriculture (Figure 7).

## Author Contributions

D.W. drafted the manuscript. R.Z., W.Z. and Y.Z. collected and organised the literature. W.Z. and T.S. participated in manuscript revision. Q.W., Z.Q.F. and Y.Q. supervised the work and finalised the manuscript. All authors read and approved the final manuscript.

## Funding

This work was funded by Chinese Academy of Tropical Agricultural Sciences for Science and Technology Innovation Team of National Tropical Agricultural Science Center (CATASCXTD202402), Project of State Key Laboratory of Tropical Crop Breeding (SKLTCBQN202514, NKLTCBCXTD24, NKLTCB‐HZ04, NKLTCB‐RC202401 and NKLTCBCXTD38), Project of Sanya Yazhou Bay Science and Technology City (SKJC‐JYRC‐2025‐63), Science and Technology Major Project of Guangxi (Guike AA23073001), Central Public‐interest Scientific Institution Basal Research Fund (163002025013, 1630052025021 and 1630052025034), National Key R&D Program of China (2022YFD2301100), Guangxi Key Laboratory of Sugarcane Genetic Improvement (21‐238‐16‐K‐02‐07) and China Agriculture Research System of MOF and MARA (CARS‐17).

## Conflicts of Interest

The authors declare no conflicts of interest.

## Data Availability

Data availability is not applicable to this article as no new data were created or analysed in this study.

## References

[pbi70542-bib-0001] Chen, F. , Y. Hu , A. Vannozzi , et al. 2017. “The WRKY Transcription Factor Family in Model Plants and Crops.” CRC Critical Reviews in Plant Sciences 36: 311–335.

[pbi70542-bib-0002] Cheng, W. , N. Wang , Y. Li , et al. 2024. “CaWRKY01‐10 and CaWRKY08‐4 Confer Pepper's Resistance to Phytophthora Capsici Infection by Directly Activating a Cluster of Defense‐Related Genes.” Journal of Agricultural and Food Chemistry 72: 11682–11693. 38739764 10.1021/acs.jafc.4c01024

[pbi70542-bib-0003] Choi, N. , J. H. Im , E. Lee , et al. 2020. “WRKY10 Transcriptional Regulatory Cascades in Rice Are Involved in Basal Defense and Xa1‐Mediated Resistance.” Journal of Experimental Botany 71: 3735–3748. 32227093 10.1093/jxb/eraa135

[pbi70542-bib-0004] Dang, F. , J. Lin , Y. Li , et al. 2023. “SlWRKY30 and SlWRKY81 Synergistically Modulate Tomato Immunity to *Ralstonia Solanacearum* by Directly Regulating *SlPR‐STH2* .” Horticultural Research 10: uhad050. 10.1093/hr/uhad050 PMC10189802 37206055

[pbi70542-bib-0005] Deslandes, L. , J. Olivier , F. Theulieres , et al. 2002. “Resistance to *Ralstonia solanacearum* in *Arabidopsis Thaliana* Is Conferred by the Recessive *RRS1‐R* Gene, a Member of a Novel Family of Resistance Genes.” Proceedings of the National Academy of Sciences of the United States of America 99: 2404–2409. 11842188 10.1073/pnas.032485099 PMC122377

[pbi70542-bib-0006] Dhatterwal, P. , N. Sharma , and M. Prasad . 2024. “Decoding the Functionality of Plant Transcription Factors.” Journal of Experimental Botany 75: 4745–4759. 38761104 10.1093/jxb/erae231

[pbi70542-bib-0007] Dong, J. , C. Chen , and Z. Chen . 2003. “Expression Profiles of the Arabidopsis WRKY Gene Superfamily During Plant Defense Response.” Plant Molecular Biology 51: 21–37. 12602888 10.1023/a:1020780022549

[pbi70542-bib-0008] Dong, Q. , D. Duan , F. Wang , et al. 2024. “The MdVQ37‐MdWRKY100 Complex Regulates Salicylic Acid Content and *MdRPM1* Expression to Modulate Resistance to Glomerella Leaf Spot in Apples.” Plant Biotechnology Journal 22: 2364–2376. 38683692 10.1111/pbi.14351 PMC11258982

[pbi70542-bib-0092] Duan, Y. , G. Yang , J. Tang , et al. 2024. “ *Ustilaginoidea virens* secreted effector UvSec117 hijacks OsWRKY31‐OsAOC module to suppress jasmonic acid‐mediated immunity in rice.” Plant Biotechnology Journal 22: 3342–3344. 39151038 10.1111/pbi.14452 PMC11606405

[pbi70542-bib-0009] Eulgem, T. , P. J. Rushton , S. Robatzek , and I. E. Somssich . 2000. “The WRKY Superfamily of Plant Transcription Factors.” Trends in Plant Science 5: 199–206. 10785665 10.1016/s1360-1385(00)01600-9

[pbi70542-bib-0010] Feng, M. , F. Augstein , A. Kareem , and C. W. Melnyk . 2024. “Plant Grafting: Molecular Mechanisms and Applications.” Molecular Plant 17: 75–91. 38102831 10.1016/j.molp.2023.12.006

[pbi70542-bib-0011] Guo, Y. , Y. Jiang , M. Wu , A. Tu , J. Yin , and J. Yang . 2024. “TaWRKY50‐TaSARK7 Module‐Mediated Cysteine‐Rich Protein Phosphorylation Suppresses the Programmed Cell Death Response to Chinese Wheat Mosaic Virus Infection.” Virology 595: 110071. 38593594 10.1016/j.virol.2024.110071

[pbi70542-bib-0012] Han, X. , L. Zhang , L. Zhao , et al. 2020. “SnRK1 Phosphorylates and Destabilizes WRKY3 to Enhance Barley Immunity to Powdery Mildew.” Plant Communications 1: 100083. 33367247 10.1016/j.xplc.2020.100083 PMC7747994

[pbi70542-bib-0013] Hao, Z. , J. Tian , H. Fang , et al. 2022. “A VQ‐Motif‐Containing Protein Fine‐Tunes Rice Immunity and Growth by a Hierarchical Regulatory Mechanism.” Cell Reports 40: 111235. 35977497 10.1016/j.celrep.2022.111235

[pbi70542-bib-0014] He, Z. , S. Webster , and S. Y. He . 2022. “Growth‐Defense Trade‐Offs in Plants.” Current Biology 32: R634–R639. 35728544 10.1016/j.cub.2022.04.070

[pbi70542-bib-0015] Hu, Q. , S. Xiao , X. Wang , C. Ao , X. Zhang , and L. Zhu . 2021. “ *GhWRKY1‐Like* Enhances Cotton Resistance to *Verticillium dahliae* via an Increase in Defense‐Induced Lignification and S Monolignol Content.” Plant Science 305: 110833. 33691967 10.1016/j.plantsci.2021.110833

[pbi70542-bib-0016] Hu, T. , X. Zhang , S. Khanal , et al. 2024. “Climate Change Impacts on Crop Yields: A Review of Empirical Findings, Statistical Crop Models, and Machine Learning Methods.” Environmental Modelling & Software 179: 106119.

[pbi70542-bib-0017] Huang, H. , X. Ma , L. Sun , et al. 2025. “SlVQ15 Recruits SlWRKY30IIc to Link With Jasmonate Pathway in Regulating Tomato Defence Against Root‐Knot Nematodes.” Plant Biotechnology Journal 23: 235–249. 39501496 10.1111/pbi.14493 PMC11672745

[pbi70542-bib-0018] Hussain, M. , X. Gao , D. Qin , X. Qin , and G. Wu . 2023. “Role of Biotic and Abiotic Factors for Sustainable Cotton Production.” In Best Crop Management and Processing Practices for Sustainable Cotton Production. IntechOpen.

[pbi70542-bib-0019] Im, J. H. , N. Choi , J. Lee , M. Y. Jung , S. R. Park , and D. J. Hwang . 2025. “Transcription Activator‐Like Effectors of *Xanthomonas oryzae* pv. Oryzae Hijack Host Transcriptional Regulation Through OsWRKYs.” Journal of Integrative Plant Biology 67: 2198–2213. 40432507 10.1111/jipb.13940 PMC12315496

[pbi70542-bib-0020] Inoue, H. , N. Hayashi , A. Matsushita , et al. 2013. “Blast Resistance of CC‐NB‐LRR Protein Pb1 Is Mediated by WRKY45 Through Protein‐Protein Interaction.” Proceedings of the National Academy of Sciences of the United States of America 110: 9577–9582. 23696671 10.1073/pnas.1222155110 PMC3677490

[pbi70542-bib-0021] Ishiguro, S. , and K. Nakamura . 1994. “Characterization of a cDNA Encoding a Novel DNA‐Binding Protein, SPF1, That Recognizes SP8 Sequences in the 5′ Upstream Regions of Genes Coding for Sporamin and β‐Amylase From Sweet Potato.” Molecular & General Genetics 244: 563–571. 7969025 10.1007/BF00282746

[pbi70542-bib-0022] Jahan, T. , M. N. Huda , K. Zhang , et al. 2025. “Plant Secondary Metabolites Against Biotic Stresses for Sustainable Crop Protection.” Biotechnology Advances 79: 108520. 39855404 10.1016/j.biotechadv.2025.108520

[pbi70542-bib-0023] Ji, S. X. , F. B. Zhang , H. D. Song , et al. 2024. “Genome‐Wide Analysis of the WRKY Family in *Nicotiana benthamiana* Reveals Key Members Regulating Lignin Synthesis and *Bemisia tabaci* Resistance.” Industrial Crops and Products 222: 119655.

[pbi70542-bib-0024] Jiang, J. , S. Ma , N. Ye , M. Jiang , J. Cao , and J. Zhang . 2017. “WRKY Transcription Factors in Plant Responses to Stresses.” Journal of Integrative Plant Biology 59: 86–101. 27995748 10.1111/jipb.12513

[pbi70542-bib-0025] Jiang, Y. , W. Zheng , J. Li , et al. 2020. “NbWRKY40 Positively Regulates the Response of *Nicotiana benthamiana* to Tomato Mosaic Virus via Salicylic Acid Signaling.” Frontiers in Plant Science 11: 603518. 33552099 10.3389/fpls.2020.603518 PMC7857026

[pbi70542-bib-0026] Jones, J. D. G. , B. J. Staskawicz , and J. L. Dangl . 2024. “The Plant Immune System: From Discovery to Deployment.” Cell 187: 2095–2116. 38670067 10.1016/j.cell.2024.03.045

[pbi70542-bib-0027] Lai, Y. , and S. Wang . 2025. “Epigenetic Regulation in Insect‐Microbe Interactions.” Annual Review of Entomology 70: 293–311. 10.1146/annurev-ento-022724-010640 39374433

[pbi70542-bib-0028] Li, M. , S. Zhao , J. Yang , et al. 2022. “Exogenous Expression of Barley *HvWRKY6* in Wheat Improves Broad‐Spectrum Resistance to Leaf Rust, *Fusarium* Crown Rot, and Sharp Eyespot.” International Journal of Biological Macromolecules 218: 1002–1012. 35872316 10.1016/j.ijbiomac.2022.07.138

[pbi70542-bib-0029] Li, Y. , X. Ma , L. D. Xiao , Y. N. Yu , and Z. H. Gong . 2025. “CaWRKY20 Negatively Regulates Plant Resistance to *Colletotrichum scovillei* in Pepper.” Plant, Cell & Environment 48: 1514–1534. 10.1111/pce.15205 39462903

[pbi70542-bib-0030] Liu, D. , J. He , Q. Li , et al. 2025. “A WRKY Transcription Factor Confers Broad‐Spectrum Resistance to Biotic Stresses and Yield Stability in Rice.” Proceedings of the National Academy of Sciences of the United States of America 122: e2411164122. 40042898 10.1073/pnas.2411164122 PMC11912400

[pbi70542-bib-0031] Ma, C. S. , B. X. Wang , X. J. Wang , et al. 2025. “Crop Pest Responses to Global Changes in Climate and Land Management.” Nature Reviews Earth and Environment 6: 264–283.

[pbi70542-bib-0032] Ma, J. , L. Wei , K. Huang , et al. 2025. “Biosynthesis of Sakuranetin Regulated by OsMPK6‐OsWRKY67‐OsNOMT Cascade Enhances Resistance to False Smut Disease.” New Phytologist 245: 1216–1231. 39611538 10.1111/nph.20308

[pbi70542-bib-0033] Mehdi, F. , Z. Cao , S. Zhang , et al. 2024. “Factors Affecting the Production of Sugarcane Yield and Sucrose Accumulation: Suggested Potential Biological Solutions.” Frontiers in Plant Science 15: 1374228. 38803599 10.3389/fpls.2024.1374228 PMC11128568

[pbi70542-bib-0034] Meng, F. , X. Zheng , J. Wang , et al. 2024. “The GRAS Protein OsDLA Involves in Brassinosteroid Signalling and Positively Regulates Blast Resistance by Forming a Module With GSK2 and OsWRKY53 in Rice.” Plant Biotechnology Journal 22: 363–378. 37794842 10.1111/pbi.14190 PMC10826986

[pbi70542-bib-0035] Meshi, T. , and M. Iwabuchi . 1995. “Plant Transcription Factors.” Plant and Cell Physiology 36: 1405–1420. 8589926

[pbi70542-bib-0036] Mi, X. , W. Li , C. Chen , et al. 2024. “GhMPK9‐GhRAF39_1‐GhWRKY40a Regulates the GhERF1b‐ and GhABF2‐Mediated Pathways to Increase Cotton Disease Resistance.” Advanced Science 11: e2404400. 38845189 10.1002/advs.202404400 PMC11304259

[pbi70542-bib-0037] Pan, X. , S. Xu , G. Cao , et al. 2025. “A Novel Peptide Encoded by a Rice Circular RNA Confers Broad‐Spectrum Disease Resistance in Rice Plants.” New Phytologist 246: 689–701. 40007179 10.1111/nph.70018

[pbi70542-bib-0038] Poosapati, S. , E. Poretsky , K. Dressano , et al. 2022. “A Sorghum Genome‐Wide Association Study (GWAS) Identifies a WRKY Transcription Factor as a Candidate Gene Underlying Sugarcane Aphid (*Melanaphis sacchari*) Resistance.” Planta 255: 37. 35020066 10.1007/s00425-021-03814-x

[pbi70542-bib-0039] Qiao, Y. , R. Xia , J. Zhai , et al. 2021. “Small RNAs in Plant Immunity and Virulence of Filamentous Pathogens.” Annual Review of Phytopathology 59: 265–288. 10.1146/annurev-phyto-121520-023514 34077241

[pbi70542-bib-0040] Ramamoorthy, R. , S. Y. Jiang , N. Kumar , P. N. Venkatesh , and S. Ramachandran . 2008. “A Comprehensive Transcriptional Profiling of the WRKY Gene Family in Rice Under Various Abiotic and Phytohormone Treatments.” Plant and Cell Physiology 49: 865–879. 18413358 10.1093/pcp/pcn061

[pbi70542-bib-0041] Ramos, R. N. , N. Zhang , D. B. Lauff , et al. 2023. “Loss‐Of‐Function Mutations in WRKY22 and WRKY25 Impair Stomatal‐Mediated Immunity and PTI and ETI Responses Against *Pseudomonas syringae* pv. *tomato* .” Plant Molecular Biology 112: 161–177. 37226022 10.1007/s11103-023-01358-0

[pbi70542-bib-0042] Reboledo, G. , A. Agorio , and I. Ponce De León . 2022. “Moss Transcription Factors Regulating Development and Defense Responses to Stress.” Journal of Experimental Botany 73: 4546–4561. 35167679 10.1093/jxb/erac055

[pbi70542-bib-0043] Rezaei, E. E. , H. Webber , S. Asseng , et al. 2023. “Climate Change Impacts on Crop Yields.” Nature Reviews Microbiology 4: 831–846.

[pbi70542-bib-0044] Rivero, R. M. , R. Mittler , E. Blumwald , and S. I. Zandalinas . 2022. “Developing Climate‐Resilient Crops: Improving Plant Tolerance to Stress Combination.” Plant Journal 109: 373–389. 10.1111/tpj.15483 34482588

[pbi70542-bib-0045] Rönspies, M. , A. Dorn , P. Schindele , and H. Puchta . 2021. “CRISPR‐Cas‐Mediated Chromosome Engineering for Crop Improvement and Synthetic Biology.” Nature Plants 7: 566–573. 33958776 10.1038/s41477-021-00910-4

[pbi70542-bib-0046] Ross, C. A. , Y. Liu , and Q. J. Shen . 2010. “The WRKY Gene Family in Rice (Oryza Sativa).” Journal of Integrative Plant Biology 49: 827–842.

[pbi70542-bib-0047] Roussin‐Léveillée, C. , D. Mackey , G. Ekanayake , R. Gohmann , and P. Moffett . 2024. “Extracellular Niche Establishment by Plant Pathogens.” Nature Reviews Microbiology 22: 360–372. 38191847 10.1038/s41579-023-00999-8 PMC11593749

[pbi70542-bib-0048] Rushton, D. L. , P. Tripathi , R. C. Rabara , et al. 2012. “WRKY Transcription Factors: Key Components in Abscisic Acid Signalling.” Plant Biotechnology Journal 10: 2–11. 21696534 10.1111/j.1467-7652.2011.00634.x

[pbi70542-bib-0049] Rushton, P. J. , I. E. Somssich , P. Ringler , and Q. J. Shen . 2010. “WRKY Transcription Factors.” Trends in Plant Science 15: 247–258. 20304701 10.1016/j.tplants.2010.02.006

[pbi70542-bib-0050] Rushton, P. J. , J. T. Torres , M. Parniske , P. Wernert , K. Hahlbrock , and I. E. Somssich . 1996. “Interaction of Elicitor‐Induced DNA‐Binding Proteins With Elicitor Response Elements in the Promoters of Parsley PR1 Genes.” EMBO Journal 15: 5690–5700. 8896462 PMC452313

[pbi70542-bib-0051] Sarris, P. F. , Z. Duxbury , S. U. Huh , et al. 2015. “A Plant Immune Receptor Detects Pathogen Effectors That Target WRKY Transcription Factors.” Cell 161: 1089–1100. 26000484 10.1016/j.cell.2015.04.024

[pbi70542-bib-0052] Savary, S. , L. Willocquet , S. J. Pethybridge , P. Esker , N. McRoberts , and A. Nelson . 2019. “The Global Burden of Pathogens and Pests on Major Food Crops.” Nature Ecology & Evolution 3: 430–439. 30718852 10.1038/s41559-018-0793-y

[pbi70542-bib-0053] Sharma, S. , A. Prasad , and M. Prasad . 2023. “Ubiquitination From the Perspective of Plant Pathogens.” Journal of Experimental Botany 74: 4367–4376. 37226440 10.1093/jxb/erad191

[pbi70542-bib-0054] Shi, X. , Y. Xiong , K. Zhang , et al. 2023. “The ANIP1‐OsWRKY62 Module Regulates Both Basal Defense and Pi9‐Mediated Immunity Against *Magnaporthe oryzae* in Rice.” Molecular Plant 16: 739–755. 36872602 10.1016/j.molp.2023.03.001

[pbi70542-bib-0055] Strader, L. , D. Weijers , and D. Wagner . 2022. “Plant Transcription Factors ‐ Being in the Right Place With the Right Company.” Current Opinion in Plant Biology 65: 102136. 34856504 10.1016/j.pbi.2021.102136 PMC8844091

[pbi70542-bib-0056] Sun, S. , S. Li , X. Zhou , and X. Yang . 2023. “WRKY1 Represses the WHIRLY1 Transcription Factor to Positively Regulate Plant Defense Against Geminivirus Infection.” PLoS Pathogens 19: e1011319. 37027442 10.1371/journal.ppat.1011319 PMC10115308

[pbi70542-bib-0057] Todeschini, A. L. , A. Georges , and R. A. Veitia . 2014. “Transcription Factors: Specific DNA Binding and Specific Gene Regulation.” Trends in Genetics 30: 211–219. 24774859 10.1016/j.tig.2014.04.002

[pbi70542-bib-0058] Wang, D. , Y. Gou , C. Yi , et al. 2025. “ScWRKY2: A Key Regulator for Smut Resistance in Sugarcane.” Plant Biotechnology Journal 23: 3667–3681. 40488671 10.1111/pbi.70186 PMC12392974

[pbi70542-bib-0059] Wang, D. , L. Wang , W. Su , et al. 2020. “A Class III WRKY Transcription Factor in Sugarcane Was Involved in Biotic and Abiotic Stress Responses.” Scientific Reports 10: 20964. 33262418 10.1038/s41598-020-78007-9 PMC7708483

[pbi70542-bib-0060] Wang, D. , W. Wang , S. Zang , et al. 2024. “Sugarcane Transcription Factor ScWRKY4 Negatively Regulates Resistance to Pathogen Infection Through the JA Signaling Pathway.” Crop Journal 12: 164–176.

[pbi70542-bib-0061] Wang, J. , L. Zhou , H. Shi , et al. 2018. “A Single Transcription Factor Promotes Both Yield and Immunity in Rice.” Science 361: 1026–1028. 30190406 10.1126/science.aat7675

[pbi70542-bib-0062] Wang, L. , D. Guo , G. Zhao , et al. 2022. “Group IIc WRKY Transcription Factors Regulate Cotton Resistance to *Fusarium Oxysporum* by Promoting GhMKK2‐Mediated Flavonoid Biosynthesis.” New Phytologist 236: 249–265. 35727190 10.1111/nph.18329

[pbi70542-bib-0063] Wang, L. , F. Liu , X. Zhang , et al. 2018. “Expression Characteristics and Functional Analysis of the *ScWRKY3* Gene From Sugarcane.” International Journal of Molecular Sciences 19: 4059. 30558233 10.3390/ijms19124059 PMC6321069

[pbi70542-bib-0064] Wang, N. , X. Fan , M. He , et al. 2022. “Transcriptional Repression of TaNOX10 by TaWRKY19 Compromises ROS Generation and Enhances Wheat Susceptibility to Stripe Rust.” Plant Cell 34: 1784–1803. 34999846 10.1093/plcell/koac001 PMC9048928

[pbi70542-bib-0065] Wang, S. , S. Han , X. Zhou , et al. 2023. “Phosphorylation and Ubiquitination of OsWRKY31 Are Integral to OsMKK10‐2‐Mediated Defense Responses in Rice.” Plant Cell 35: 2391–2412. 36869655 10.1093/plcell/koad064 PMC10226564

[pbi70542-bib-0066] Wang, Y. , X. Wang , J. Fang , et al. 2023. “VqWRKY56 Interacts With VqbZIPC22 in Grapevine to Promote Proanthocyanidin Biosynthesis and Increase Resistance to Powdery Mildew.” New Phytologist 237: 1856–1875. 36527243 10.1111/nph.18688

[pbi70542-bib-0067] Wu, J. , Y. Zhang , F. Li , et al. 2024. “Plant Virology in the 21st Century in China: Recent Advances and Future Directions.” Journal of Integrative Plant Biology 66: 579–622. 37924266 10.1111/jipb.13580

[pbi70542-bib-0068] Xiao, S. , Y. Ming , Q. Hu , et al. 2023. “GhWRKY41 Forms a Positive Feedback Regulation Loop and Increases Cotton Defence Response Against *Verticillium dahliae* by Regulating Phenylpropanoid Metabolism.” Plant Biotechnology Journal 21: 961–978. 36632704 10.1111/pbi.14008 PMC10106861

[pbi70542-bib-0069] Xie, Q. , W. Dong , M. Wang , et al. 2025. “ *BpWRKY6* Regulates Insect Resistance by Affecting Jasmonic Acid and Terpenoid Synthesis in *Betula platyphylla* .” Plant Biotechnology Journal 23: 3682–3696. 40493356 10.1111/pbi.70169 PMC12392942

[pbi70542-bib-0070] Xie, W. , Y. Ke , J. Cao , S. Wang , and M. Yuan . 2021. “Knock Out of Transcription Factor WRKY53 Thickens Sclerenchyma Cell Walls, Confers Bacterial Blight Resistance.” Plant Physiology 187: 1746–1761. 34618083 10.1093/plphys/kiab400 PMC8566205

[pbi70542-bib-0071] Xu, H. , K. A. Watanabe , L. Zhang , and Q. J. Shen . 2016. “WRKY Transcription Factor Genes in Wild Rice *Oryza nivara* .” DNA Research 23: 311–323. 27345721 10.1093/dnares/dsw025 PMC4991837

[pbi70542-bib-0072] Xu, Y. P. , H. Xu , B. Wang , and X. D. Su . 2019. “Crystal Structures of N‐Terminal WRKY Transcription Factors and DNA Complexes.” Protein & Cell 11: 208–213. 10.1007/s13238-019-00670-0 PMC7026264 31734872

[pbi70542-bib-0073] Yamasaki, K. , T. Kigawa , M. Inoue , et al. 2005. “Solution Structure of an Arabidopsis WRKY DNA Binding Domain.” Plant Cell 17: 944–956. 15705956 10.1105/tpc.104.026435 PMC1069710

[pbi70542-bib-0074] Yang, L. , S. Fang , L. Liu , et al. 2025. “WRKY Transcription Factors: Hubs for Regulating Plant Growth and Stress Responses.” Journal of Integrative Plant Biology 67: 488–509. 39815727 10.1111/jipb.13828

[pbi70542-bib-0075] Yang, M. , Y. Wang , C. Chen , et al. 2024. “Transcription Factor WRKY75 Maintains Auxin Homeostasis to Promote Tomato Defense Against *Pseudomonas syringae* .” Plant Physiology 195: 1053–1068. 38245840 10.1093/plphys/kiae025

[pbi70542-bib-0076] Yang, S. , W. Cai , L. Shen , et al. 2022. “A CaCDPK29‐CaWRKY27b Module Promotes CaWRKY40‐Mediated Thermotolerance and Immunity to *Ralstonia solanacearum* in Pepper.” New Phytologist 233: 1843–1863. 34854082 10.1111/nph.17891

[pbi70542-bib-0077] Yang, Y. , H. Huang , Z. Xin , et al. 2025. “Functional Characterization of TaWRKY254 in Salt Tolerance Based on Genome‐Wide Analysis of the WRKY Gene Family in Wheat Core Parent Zhou8425B.” Plant Science 357: 112540. 40320010 10.1016/j.plantsci.2025.112540

[pbi70542-bib-0078] Yuan, H. , M. Cheng , F. Fan , et al. 2024. “OsGRF6‐OsYUCCA1/OsWRKY82 Signaling Cascade Upgrade Grain Yield and Bacterial Blight Resistance in Rice.” Advanced Science 11: e2407733. 39441559 10.1002/advs.202407733 PMC11633520

[pbi70542-bib-0079] Zandalinas, S. I. , F. B. Fritschi , and R. Mittler . 2021. “Global Warming, Climate Change, and Environmental Pollution: Recipe for a Multifactorial Stress Combination Disaster.” Trends in Plant Science 26: 588–599. 33745784 10.1016/j.tplants.2021.02.011

[pbi70542-bib-0080] Zandalinas, S. I. , and R. Mittler . 2022. “Plant Responses to Multifactorial Stress Combination.” New Phytologist 234: 1161–1167. 35278228 10.1111/nph.18087

[pbi70542-bib-0081] Zang, S. , D. Wang , L. Qin , et al. 2025. “ScWRKY6 Interacts With ScSAG39 to Regulate Immune Homeostasis by Transcriptional Control of ScPR1.” Plant Biotechnology Journal. 10.1111/pbi.70444. PMC12946490 41199635

[pbi70542-bib-0082] Zhang, Q. , Q. Xu , N. Zhang , et al. 2024. “A Maize WAK‐SnRK1α2‐WRKY Module Regulates Nutrient Availability to Defend Against Head Smut Disease.” Molecular Plant 17: 1654–1671. 39360383 10.1016/j.molp.2024.09.013

[pbi70542-bib-0083] Zhang, S. , L. Dong , X. Zhang , et al. 2023. “The Transcription Factor *GhWRKY70* From *Gossypium hirsutum* Enhances Resistance to Verticillium Wilt via the Jasmonic Acid Pathway.” BMC Plant Biology 23: 141. 36915047 10.1186/s12870-023-04141-x PMC10012446

[pbi70542-bib-0084] Zhang, Y. , and L. Wang . 2005. “The WRKY Transcription Factor Superfamily: Its Origin in Eukaryotes and Expansion in Plants.” BMC Evolutionary Biology 5: 1. 15629062 10.1186/1471-2148-5-1 PMC544883

[pbi70542-bib-0085] Zhang, Z. , C. Jiang , C. Chen , et al. 2023. “VvWRKY5 Enhances White Rot Resistance in Grape by Promoting the Jasmonic Acid Pathway.” Horticultural Research 10: uhad172. 10.1093/hr/uhad172 PMC10569242 37841502

[pbi70542-bib-0086] Zhao, Y. Q. , C. Sun , K. D. Hu , et al. 2025. “A Transcription Factor SlWRKY71 Activated the H_2_S Generating Enzyme *SlDCD1* Enhancing the Response to *Pseudomonas syringae* pv DC3000 in Tomato Leaves.” New Phytologist 246: 262–279. 39887348 10.1111/nph.20431

[pbi70542-bib-0087] Zhao, Z. , R. Wang , W. Su , et al. 2024. “A Comprehensive Analysis of the WRKY Family in Soybean and Functional Analysis of GmWRKY164‐GmGSL7c in Resistance to Soybean Mosaic Virus.” BMC Genomics 25: 620. 38898399 10.1186/s12864-024-10523-8 PMC11188170

[pbi70542-bib-0088] Zheng, C. , J. Zhou , X. Yuan , et al. 2024. “Elevating Plant Immunity by Translational Regulation of a Rice WRKY Transcription Factor.” Plant Biotechnology Journal 22: 1033–1048. 37997501 10.1111/pbi.14243 PMC10955491

[pbi70542-bib-0089] Zheng, J. , F. Liu , C. Zhu , et al. 2019. “Identification, Expression, Alternative Splicing and Functional Analysis of Pepper WRKY Gene Family in Response to Biotic and Abiotic Stresses.” PLoS One 14: e0219775. 31329624 10.1371/journal.pone.0219775 PMC6645504

[pbi70542-bib-0090] Zheng, X. , B. Koopmann , B. Ulber , and A. V. Tiedemann . 2020. “A Global Survey on Diseases and Pests in Oilseed Rape‐Current Challenges and Innovative Strategies of Control.” Frontiers in Agronomy 2: 590908.

[pbi70542-bib-0091] Zhou, M. , H. Wang , X. Yu , et al. 2024. “Transcription Factors VviWRKY10 and VviWRKY30 Co‐Regulate Powdery Mildew Resistance in Grapevine.” Plant Physiology 195: 446–461. 38366578 10.1093/plphys/kiae080

